# Finding Markers That Make a Difference: DNA Pooling and SNP-Arrays Identify Population Informative Markers for Genetic Stock Identification

**DOI:** 10.1371/journal.pone.0082434

**Published:** 2013-12-16

**Authors:** Mikhail Ozerov, Anti Vasemägi, Vidar Wennevik, Rogelio Diaz-Fernandez, Matthew Kent, John Gilbey, Sergey Prusov, Eero Niemelä, Juha-Pekka Vähä

**Affiliations:** 1 Kevo Subarctic Research Institute, University of Turku, Turku, Finland; 2 Department of Biology, Division of Genetics and Physiology, University of Turku, Turku, Finland; 3 Research group Population Genetics and Ecology, Institute of Marine Research, Bergen, Norway; 4 Department of Animal and Aquacultural Sciences, Centre for Integrative Genetics (CIGENE), Norwegian University of Life Sciences, Ås, Norway; 5 Freshwater Laboratory, Marine Scotland, Faskally, Pitlochry, United Kingdom; 6 Freshwater Resources Laboratory, Knipovitch Polar Research Institute of Marine Fisheries and Oceanography, Murmansk, Russia; 7 Finnish Game and Fisheries Research Institute, Oulu, Finland; 8 Department of Aquaculture, Institute of Veterinary Medicine and Animal Science, Estonian University of Life Sciences, Tartu, Estonia; Aberystwyth University, United Kingdom

## Abstract

Genetic stock identification (GSI) using molecular markers is an important tool for management of migratory species. Here, we tested a cost-effective alternative to individual genotyping, known as allelotyping, for identification of highly informative SNPs for accurate genetic stock identification. We estimated allele frequencies of 2880 SNPs from DNA pools of 23 Atlantic salmon populations using Illumina SNP-chip. We evaluated the performance of four common strategies (global *F*_ST_, pairwise *F*_ST_, *Delta* and outlier approach) for selection of the most informative set of SNPs and tested their effectiveness for GSI compared to random sets of SNP and microsatellite markers. For the majority of cases, SNPs selected using the outlier approach performed best followed by pairwise *F*_ST_ and *Delta* methods. Overall, the selection procedure reduced the number of SNPs required for accurate GSI by up to 53% compared with randomly chosen SNPs. However, GSI accuracy was more affected by populations in the ascertainment group rather than the ranking method itself. We demonstrated for the first time the compatibility of different large-scale SNP datasets by compiling the largest population genetic dataset for Atlantic salmon to date. Finally, we showed an excellent performance of our top SNPs on an independent set of populations covering the main European distribution range of Atlantic salmon. Taken together, we demonstrate how combination of DNA pooling and SNP arrays can be applied for conservation and management of salmonids as well as other species.

## Introduction

The use of molecular markers for determination of an individual’s origin is an important tool in the management and conservation of domestic and wild species [1]. Individual genetic assignment has also been used in forensic cases to detect illegal trade and translocation of animals [2], illegal harvesting [1], and source of origin of escaped domesticated animals [3], [4]. Assigning individuals to populations of origin, also known as genetic stock identification (GSI), has been particularly important management tool in salmonid fishes [5].

Due to their high variability and availability, microsatellites or short tandem repeats (STR), have been the markers of choice for GSI for nearly two decades [5], [6]. However, the numerous advantages of single nucleotide polymorphism (SNP) markers such as high abundance, processing efficiency, ease of scoring and standardizing among laboratories make SNPs attractive for individual genetic assignment studies [5]. On the other hand, due to their bi-allelic nature, the power of single SNP loci is limited, requiring a larger number of independent loci in comparison to STRs. To overcome low average assignment power of SNPs compared to multi-allelic loci, selecting a small subset of highly informative loci from a large number of SNPs has been proposed [1], [7], [8]. However, initial screening for highly informative markers from among thousands of SNPs in multiple populations is expensive.

To overcome the high cost of large-scale SNP genotyping, determination of allele frequencies from pooled DNA, i.e., ‘allelotyping’, has been suggested as a cost-effective alternative for obtaining reliable allele frequency information for thousands of SNPs [9], [10]. These studies have demonstrated high accuracy and repeatability of DNA pooling approach, reducing costs up to 100 fold, depending on the number of samples [11]. Allelotyping has also allowed efficient detection of genes associated with numerous traits and diseases [12], [13]. Since allelotyping allows detection of markers with large between-group allele frequency differences [14], this approach can be applied to identify population-informative markers, i.e., a small set of powerful markers that enables accurate genetic stock identification.

For the past decade GSI has been an invaluable tool for the management and conservation of Atlantic salmon populations, enabling estimation of relative contributions of various populations in mixed stock fisheries [15] as well as identifying the population of origin of individual fish [16]. For example, these methods are used for detecting population specific migration patterns [17], estimating the proportion of farm escapees in salmon fisheries [4], [18] and identification of non-native hatchery-bred individuals in wild populations [19].

Here we tested the feasibility of combining DNA pooling and SNP arrays for identification of highly informative SNPs for accurate GSI in Atlantic salmon, focusing on individual assignment. We estimated allele frequencies of 2880 SNPs from DNA pools of 23 salmon populations using an Atlantic salmon Illumina SNP-chip. We compared the performance of four common approaches (global *F*_ST_, pairwise *F*_ST_, *Delta* and outlier) to identify the most informative SNPs. We subsequently evaluated the effects of specific population dataset and number of SNPs on individual assignment. We compared the performance of the top SNPs against 31 STR loci. We also tested the combined power of existing STR panels with the most informative SNPs. Finally, we compiled the largest population genetic dataset for Atlantic salmon to date, both in terms of geographic coverage and the number of samples, by merging our data with published data [9], [20] and validated the performance of our top SNPs on an independent set of populations covering the main European distribution range.

## Materials and Methods

### Samples & DNA Pooling

**Figure 1:**
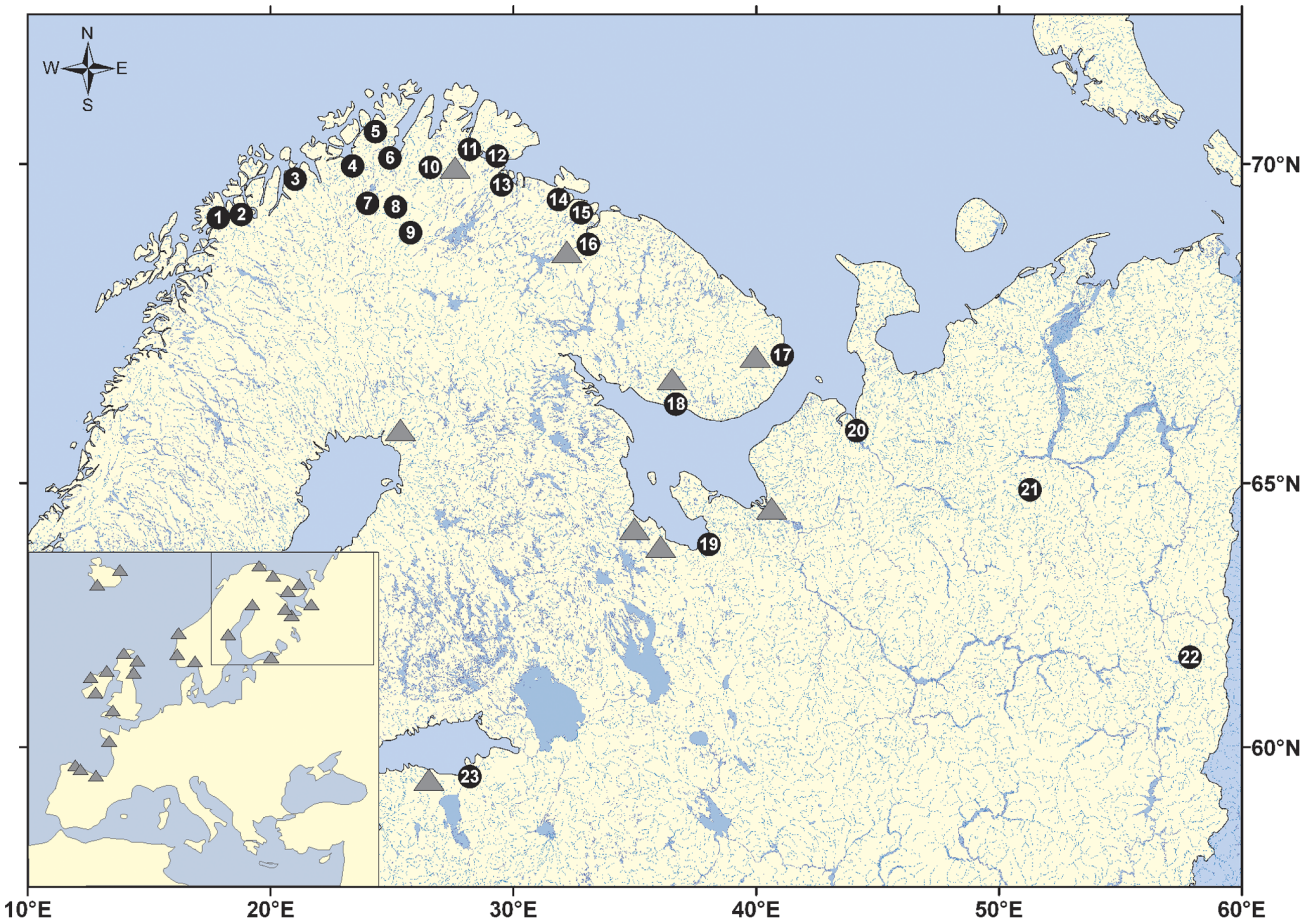
Map indicating sampling locations of the studied populations. See Table 1 for population names. European Atlantic salmon samples [20] used for validation of top ranked SNPs are indicated as filled triangles.

**Figure 2:**
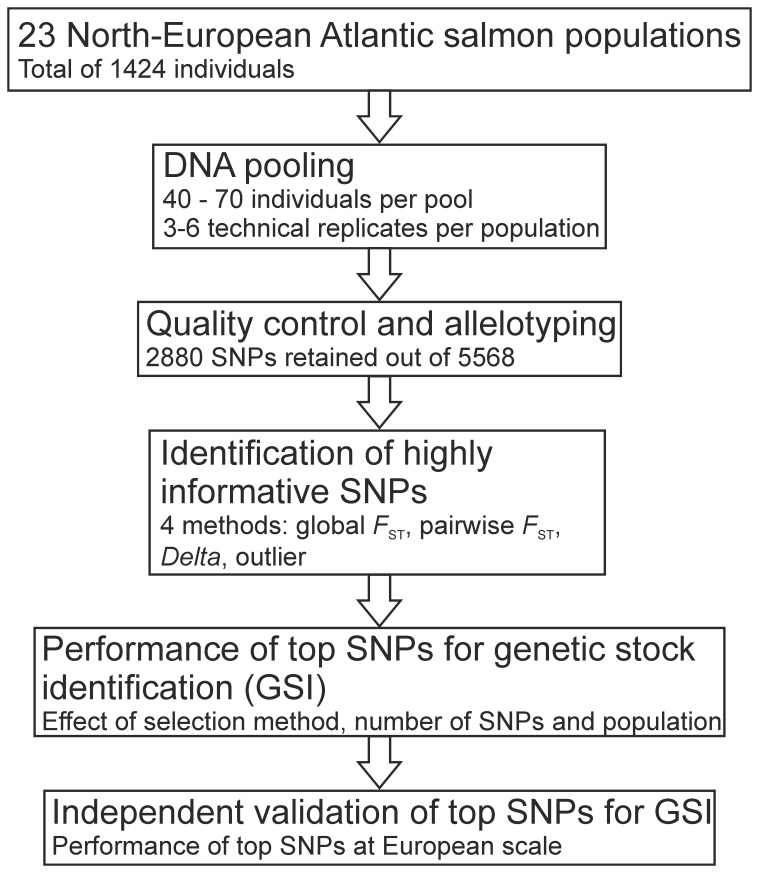
Workflow diagram indicating the main steps of the analyses.

In total, 1424 individuals were collected from 23 Atlantic salmon populations spawning in the rivers along the Norwegian and Russian north-west and Baltic Sea coasts between 17°E and 57°E (Fig. 1). Salmon juveniles were collected by electrofishing, sacrificed by decapitation and a tissue sample of each individual stored immediately in 70% ethanol. The permits for sample collection were issued by: 1) Federal Agency for Fisheries (Russia), 2) County Governor of Finnmark and Troms (Norway), 3) Center for Economic Development, Transport and the Environment (Finland), and 4) Ministry of Environment (Estonia). As the fish were sacrificed immediately after sampling and no experiments with living fish were performed the approval of ethics committee was not required (EU directive 2010/63/EU, Russian Federation government regulation 2009/921, Norwegian Animal Welfare Act 19/06/2009). This dataset included samples from 14 populations studied by Ozerov et al. [9] (Table 1). Similar to our previous work [9], equimolar (10 ng/ul) DNA extracts from 40 to 70 individuals were pooled to provide from three to six technical replicates for each population sample (Table 1, Fig. 2). The pooled DNA samples were analyzed in the Center for Integrative Genetics (CIGENE, Norway) using an Illumina infinium assay (Illumina, San Diego, CA, USA) and version 2 of the Atlantic salmon SNP-chip [20], [21] carrying probes for 5568 SNP markers. Among our study populations, 3928 of those SNP loci were bi-allelic and were further analyzed in this study. The raw SNP data were analyzed using Genotyping module v. 1.9.4 (Genome Studio software v. 2011.1, Illumina Inc.). In addition, the same 23 populations used for DNA pool construction were individually genotyped using 31 commonly applied [17], [22] STR markers (Table 1, Table S1 in Appendix S1). The STR data were analyzed and the genotypes were scored with Genemapper 4.1 software (Applied Biosystems).

**Table 1:** Information about populations included in the datasets used for SNP selection and their geographic locations.

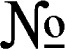	Population	Coordinates	*N*_SNP_	*N*_STR_	*H*_E_ SNPs	*H*_E_ STRs	Population dataset		
							I	II	III
	*Norwegian Sea*								
1	Laukhelle*	69°13'N 17°50'E	42 (4)	42	0.35	0.73	x	x	x
2	Målselva	69°13'N 18°29'E	70 (3)	70	0.34	0.72	x	x	
3	Reisa	69°46'N 21°00'E	70 (3)	70	0.31	0.69	x	x	x
4	Alta*	69°58'N 23°22'E	70 (3)	65	0.32	0.69	x	x	
5	Repparfjordelv*	70°26'N 24°19'E	69 (4)	67	0.35	0.73	x	x	x
	*Western Barents Sea*								
6	Lakselva*	70°04'N 24°55'E	67 (5)	67	0.32	0.68	x	x	
7	Iesjoki (Teno/Tana)	69°26'N 24°59'E	70 (3)	70	0.32	0.71	x	x	
8	Karasjoki (Teno/Tana)*	69°23'N 25°09'E	70 (4)	63	0.33	0.70	x	x	
9	Inarijoki (Teno/Tana)*	69°00'N 25°46'E	67 (4)	67	0.33	0.70	x	x	
10	Yläköngäs (Teno/Tana)	69°57'N 26°34'E	58 (3)	58	0.32	0.71	x	x	x
11	Tana Bru (Teno/Tana)*	70°12'N 28°11'E	60 (2)	59	0.34	0.70	x	x	
12	Vestre Jakobselv*	70°06'N 29°19'E	70 (4)	59	0.33	0.72	x	x	
13	Neiden*	69°42'N 29°31'E	63 (4)	63	0.33	0.72	x	x	x
14	Titovka*	69°30'N 31°58'E	70 (4)	67	0.35	0.74	x	x	
15	Ura*	69°16'N 32°48'E	44 (3)	44	0.34	0.73	x	x	x
16	Kola*	68°52'N 33°01'E	70 (6)	70	0.33	0.74	x	x	
	*White Sea*								
17	Ponoi*	66°58'N 41°16'E	70 (4)	70	0.33	0.72	x		
18	Varzuga*	66°12'N 36°57'E	70 (4)	70	0.31	0.70	x		
19	Onega	63°54'N 38°00'E	70 (3)	70	0.25	0.65	x		
20	Mezen Pizhma	65°53'N 44°08'E	48 (3)	48	0.28	0.68	x		
	*Eastern Barents Sea*								
21	Pechora Pizhma	64°52'N 51°16'E	48 (3)	48	0.26	0.67	x		
22	Pechora Unya	61°47'N 57°52'E	48 (3)	48	0.24	0.64	x		
	*Baltic Sea*								
23	Narva	59°23'N 28°12'E	40 (3)	40	0.23	0.64	x		
	***Total pooled (SNPs)***		**1424**						
	***Total individual (STRs)***			**1395**					

*N*_SNP_ – number of individuals included in the pools and number of technical replicates (in brackets) for SNP-chip analysis, *N*_STR_ – number of samples for individual genotyping using 31 STRs, *H*_E_ – overall expected heterozygosity for SNPs and STRs.

* Data from Ozerov et al. [9].

### Allele Frequency Estimation

Allele frequencies for 23 populations were estimated from DNA pools comparing pool-specific value of theta with the reference values of theta derived from 300 Atlantic salmon specimens genotyped by CIGENE [9]. Briefly, the raw color signal data from 2 alternate alleles is converted into a theta value which ranges from 0 to 1. In theory, an individual homozygous for an allele would have a theta value close to 0 or 1, and a value of 0.5 would indicate a heterozygous genotype. However, in reality a SNP’s theta for genotype clusters (AA, AB and BB) vary from theoretical values of 0, 0.5 and 1. Therefore, for estimation of allele frequency in a pooled sample, the theta value for each SNP is compared to the mean theta values for AA, AB and BB genotypes calculated by genotyping of individual samples applying correction algorithm, method 2 in [23].

Stringent quality control filters resulted in selection of 2880 bi-allelic SNPs showing low error rates (variation of theta among technical replicates ≤0.02) compared to information content [9]. Population-specific allele frequencies for each SNP were estimated as a mean over 3–6 technical replicates (Appendix S2).

### Population Relationships and within Population Diversity

In order to infer the genetic relationships among populations, pairwise *D*_A_ [24] distances and pairwise *F*_ST_ were calculated from allele frequency estimates derived from allelotyping of 2880 SNPs with the PowerMarker v3.25 software package [25]. The *D*_A_ genetic distances were used to construct neighbor-joining trees with 1000 bootstrap replicates. The same approach was applied for the 31 STR markers. Consensus dendrograms were constructed separately for SNPs and STRs by using the program SplitsTree4 v4.11.3 [26]. Similarly, PowerMarker v3.25 [25] was used to estimate expected heterozygosity (*H*_E_) of populations over all SNP and STR loci.

### Selection of the most Informative SNPs

We evaluated four different methods for identification of the most informative set of SNPs for GSI: *Delta* [27], global *F*_ST_, pairwise *F*_ST_ [28] and the outlier approach [29]. The estimate of allele-frequency differential, i.e., *Delta*, is one of the straightforward ways to evaluate the information content of a SNP. For a bi-allelic marker, like SNP, the *Delta* value is estimated as |*p*A_i_ - *p*A_j_|, where *p*A_i_ and *p*A_j_ are the frequencies of allele A in the i^th^ and j^th^ populations, respectively. *Delta* value for each SNP marker was estimated as the mean across all pair-wise comparisons of 23 populations. Another common criterion for selecting the most informative loci is the population differentiation measure *F*_ST_: the unbiased estimates of *F*_ST_ were first calculated over all populations (global *F*_ST_) and on a pairwise basis (pairwise *F*_ST_). SNPs in the upper quartile of distribution of divergence values were classified as markers having “high level” of genetic differentiation (Fig. S1 in Appendix S1). For the first three approaches (i.e., *Delta*, global *F*_ST_ and pairwise *F*_ST_), the top 300 unlinked SNPs (>1 cM distance from each other) were selected for subsequent analyses.

For identification of SNPs deviating from the neutral expectations (outliers), a Bayesian likelihood method was used, implemented in Bayescan 2.01 [29]. The method provides posterior odds (PO) as the ratio of the posterior probabilities indicating how much more likely the model with selection is compared to the neutral model. Posterior odd values between 10 and 32 (log_10_(PO) = 1–1.5) are considered as strong evidence of selection, between 32 and 100 (log_10_(PO) = 1.5–2) – as very strong, and PO above 100 (log_10_(PO) >2) are viewed as decisive evidence of selection [1], [29]. Depending on population dataset, 35–111 unlinked outliers (>1 cM distance from each other) potentially influenced by divergent selection [30] were identified and used for subsequent analyses.

To estimate how the number of SNPs affect the performance of GSI, subsets of the top 25, 50, 75, 100, 125, 150, 200, 250 and 300 SNP loci were selected for each of the four ranking approaches. In addition, we also chose similarly-sized subsets of random SNPs (i.e. 25, 50, 75, 100, 125, 150, 200, 250 and 300 SNP loci). To evaluate the effect of populations on the selection of top SNPs we tested the overall assignment power of the most informative SNPs identified using three different population datasets. The first dataset included all 23 populations (dataset I); the second set consisted of 16 populations (1–16) excluding the easternmost and Baltic salmon (dataset II); and finally, the third set consisted of six populations (1, 3, 5, 11, 13, 15) evenly distributed across the Norwegian and the Western Barents seas coasts (dataset III; Table 1).

### Performance of Top SNPs and STRs for GSI

As the methods applied for GSI require genotype data rather than allele frequency estimates, the multilocus genotypes were simulated from the allele frequency estimates assuming Hardy-Weinberg and linkage equilibrium using bespoke software (see Appendix S3 for the code). For each subset of SNP loci, 100 multilocus genotypes per population were simulated as a baseline sample. Another 500 genotypes per population for the mixed stock fishery sample were simulated for each SNP subset using ONCOR [31]. A similar approach was applied to the STR data.

The assignment of individuals in a mixture to baseline populations was performed by using ONCOR [31]. This approach estimates a probability that an individual (of unknown origin) belongs to a baseline population by assessing estimates of the genotype frequencies in each baseline population [32] and an estimate of the stock composition of the fishery [31]. In ONCOR, the simulated mixed stock fishery sample was tested against baseline data set along with the “Assign individuals to the baseline population” option to assign each fish. All individuals were assigned irrespective of precision. As the software used was specifically developed for analyzing samples of individuals the influence of the distribution of genotypes in the sample being tested was examined using mixtures of fish with varying compositions. It was found that at the levels of differentiation observed here these compositions had only minor influence on the assignment results (see Table S2 in Appendix S1). In addition to SNPs, performance was evaluated for 31 STRs. We also evaluated the performance of a 31-locus STR panel combined with different numbers of SNPs (1, 2, 5, 10, 25, 50 and 100 top-ranked loci). We estimated the number of STR or SNP alleles required to achieve 80%, 90% and 95% correct assignments for each ranking approach and population dataset. We made these estimates by fitting a non-linear regression model to the curves of correct assignment percentage against cumulative number of markers. An exponential regression model (*y* = exp(*a*+*b*/*x*)) was found to best fit the data.

### Analysis of the Independent Dataset

To evaluate the usefulness of our approach, and the effectiveness of our best SNPs for GSI in different sets of populations, independent validation was performed on the Atlantic salmon individuals genotyped by Bourret et al. [20], DRYAD entry doi:10.5061/dryad.gm367. Specifically, we evaluated the performance of our top-ranked loci (dataset II, pairwise *F*_ST_ selection approach, 25 to 150 SNPs) for GSI on 26 European anadromous populations ranging from Spain (Narcea) to Russia (Severnaja Dvina). Compared to our data, only three populations studied by Bourrret et al. [20] originated from the river systems included in both datasets (Tana/Teno, Ponoi, Varzuga). Additionally, we tested the reliability of the allelotyping approach by combining the allele frequency estimates from DNA pools of 23 populations with the 38-population dataset of Bourret et al. [20] which consisted of individual genotypes.

## Results

### Genetic Diversity and Differentiation: SNPs vs. STRs

**Figure 3:**
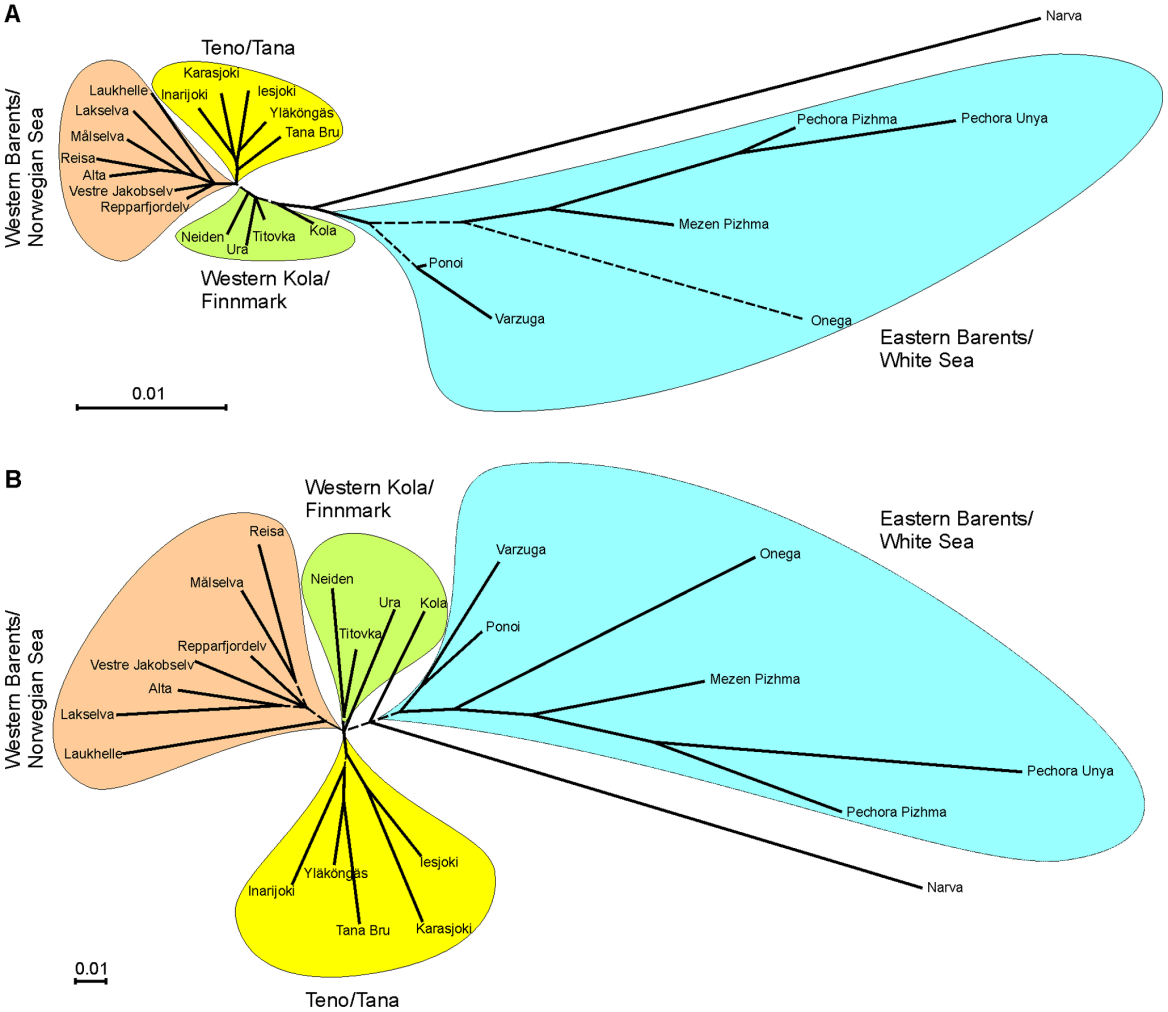
Genetic relationships among 23 Atlantic salmon populations in northern Europe. Neighbour-joining dendrogram is based on Nei’s *D*_A_ genetic distances estimated using (A) 2880 SNPs and (B) 31 STR markers. Distinct population groups are colored. The branches with bootstrap value support <80% are drawn as dashed.

As expected, the genetic diversity of SNPs over all populations was significantly lower compared to STRs (median SNP*_H_*_e_ = 0.36 *vs*. median STR*_H_*_e_ = 0.77, Mann-Whitney U-test, *P*<0.001). The genetic diversity (*H*_E_) of populations over all SNP loci ranged from 0.23 to 0.35, whereas for STR data *H*_E_ estimates were higher, ranging from 0.64 to 0.74 (Table 1). However, genetic diversity estimates within populations (*H*_E_) were significantly correlated between the two marker types (*Pearson’s r* = 0.93, *P*<0.0001). Pairwise population differentiation (*F*_ST_) estimates over 2880 SNPs varied from 0.01 (Titovka *vs*. Ura) to 0.30 (Narva *vs*. Pechora Unya), whereas mean pairwise *F*_ST_ values over 31 STRs ranged from 0.02 to 0.20 for the same population pairs (Table S3 in Appendix S1). Similar to genetic diversity, genetic divergence of the populations (mean pairwise *F*_ST_) was significantly correlated between SNPs and STRs (*Mantel’s r*_xy = _0.94, *P*<0.0001). Thus, both marker classes revealed very similar population genetic structuring as illustrated by neighbor-joining trees (Fig. 3). However, the level of differentiation of SNPs over all populations was significantly higher than that of STRs (median SNP*_F_*_st_ = 0.077 *vs*. median STR*_F_*_st_ = 0.055, Mann-Whitney U-test, *P*<0.001).

### Identification of the most Informative SNPs

**Figure 4:**
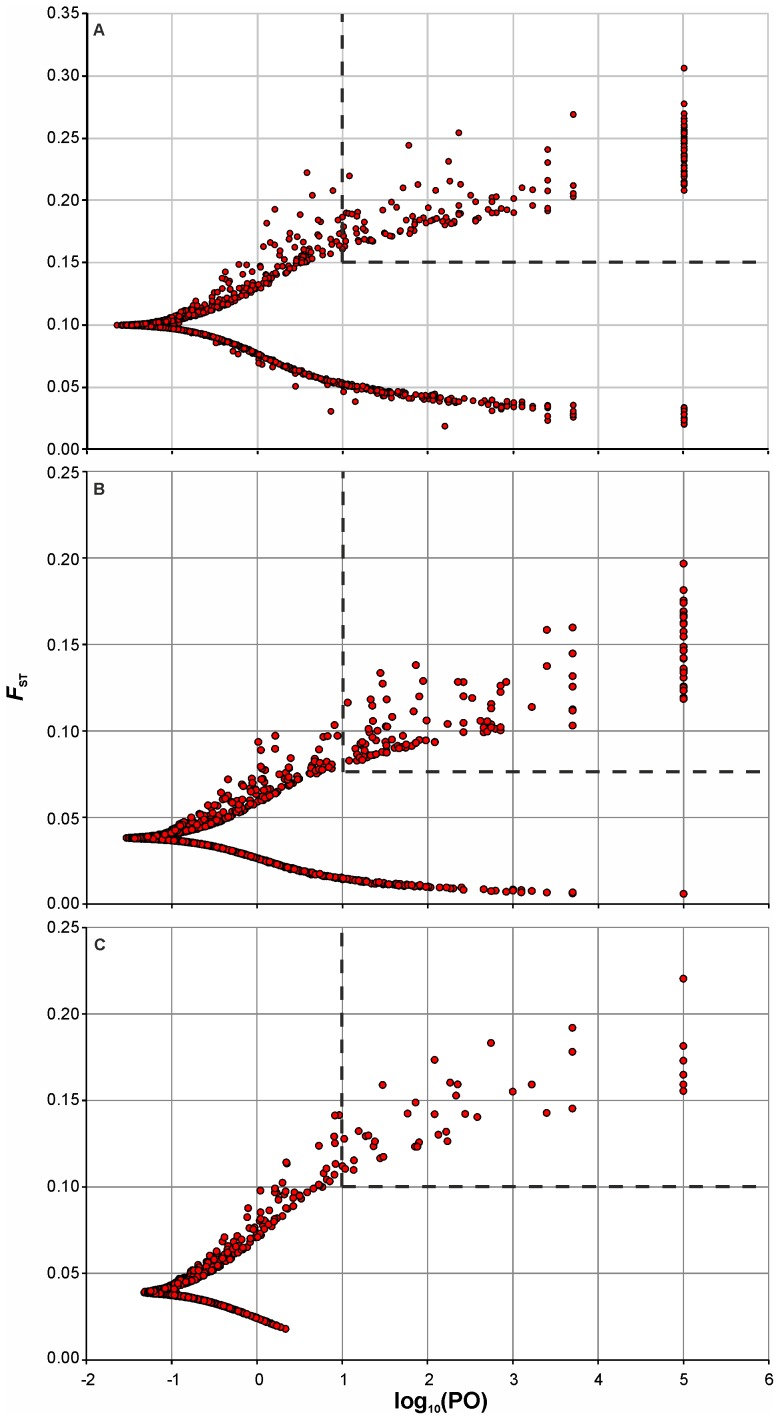
Identification of outlier loci using a model-based genome scan approach. (A) population dataset I; (B) population dataset II; (C) population dataset III. Each SNP locus (filled circle) is represented by the level of genetic differentiation (*F*_ST_) and log_10_(PO) of being under selection. Outlier loci potentially under divergent selection are inside dashed rectangle.

As expected, only a small proportion of SNPs (out of 2880) exhibited high levels of genetic differentiation estimated using three measures (global *F*_ST_, pairwise *F*_ST_ and *Delta*, Appendix S4, Fig. S1 in Appendix S1). The outlier test for population datasets I, II and III identified putative signs of divergent selection (log_10_(PO) >1, *q* <0.05) at 141, 120 and 41 SNPs, respectively (Fig. 4). However, as several SNPs formed tightly linked groups (<1 cM), a total of 111, 95 and 35 unlinked outlier SNPs were retained for subsequent analysis for population datasets I, II and III, respectively.

**Figure 5:**
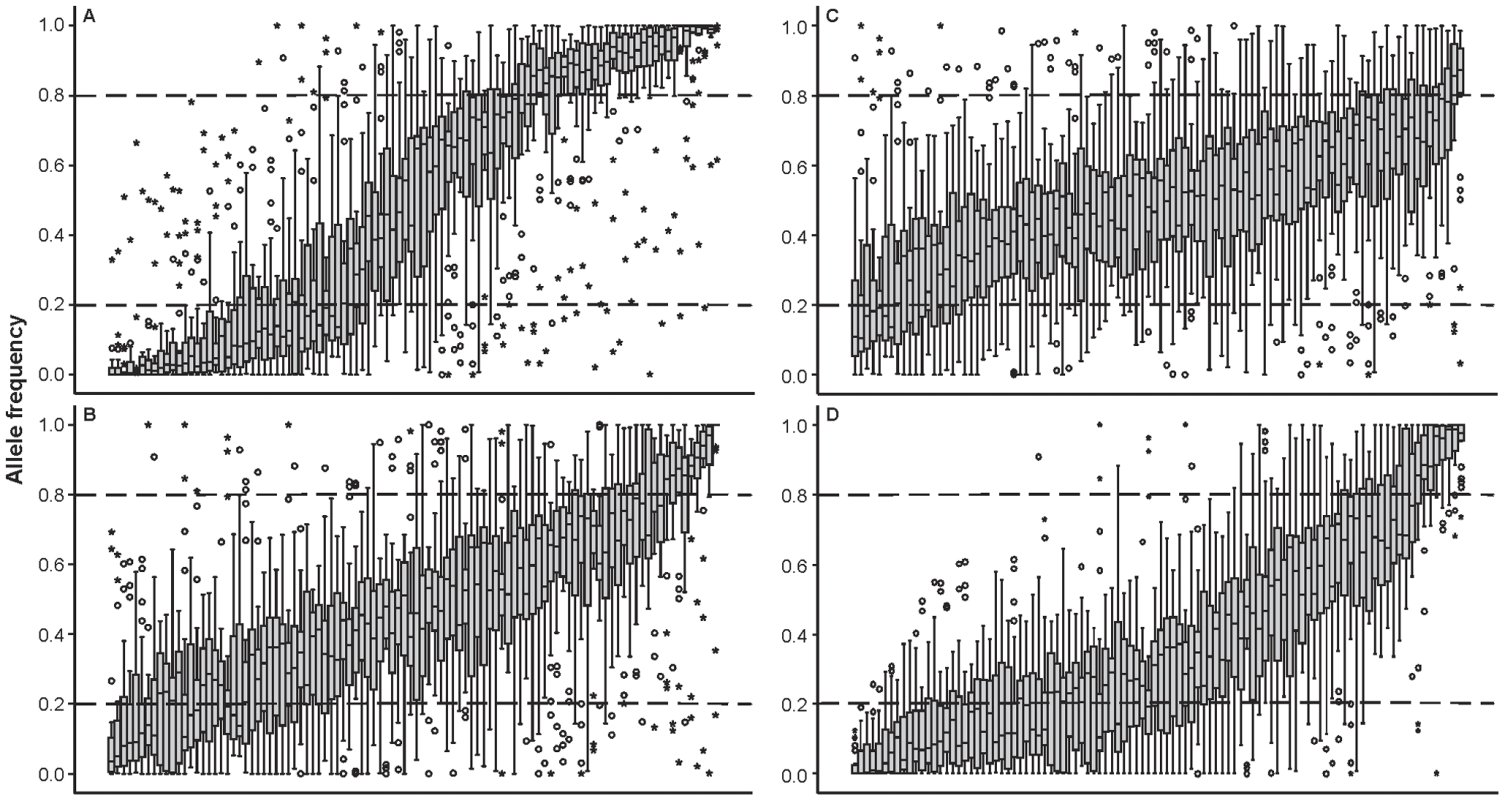
Distribution of allele frequencies for the 100 top-ranked SNPs. Allele frequencies of the 100 top-ranked SNPs in 23 populations identified using four selection approaches: (A) global *F*_ST_; (B) pairwise *F*_ST_; (C) *Delta*, and (D) outlier. Horizontal line, grey rectangle, whiskers, open circles, and stars indicate median, 25th and 75th quartiles, non-outlier range, outliers and extreme outliers, respectively.

The comparison of allele frequency distributions of 100 top SNPs ranked using *Delta* or pairwise *F*_ST_ showed that these two approaches identified loci with a wide range of allele frequencies among populations (Fig. 5). A similar pattern was evident for outliers, with a slightly higher proportion of loci (42%) showing marginal allele frequency distributions (close to 0 or 1). In contrast, the allele frequencies of a majority of the SNPs (69%) ranked using global *F*_ST_ measure were biased towards 0 or 1 with relatively few loci exhibiting intermediate allele frequencies.

**Figure 6:**
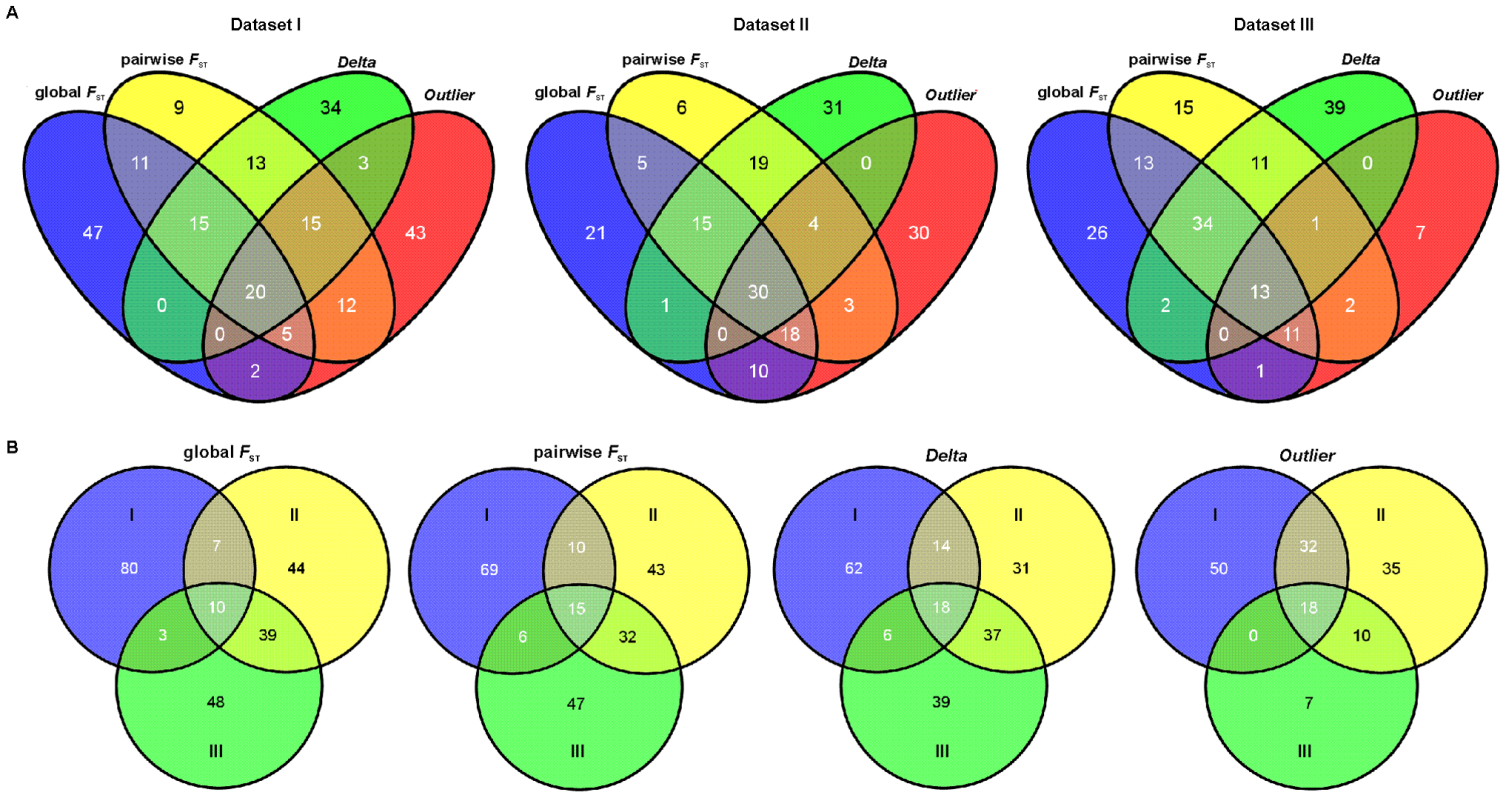
SNP overlap among different ranking approaches and population datasets. (A) Venn diagrams showing the extent of overlap among four approaches (global F_ST_, pairwise F_ST_, *Delta* and outlier) for three population datasets. (B) Venn diagrams showing the extent of overlap among three population datasets for four ranking approaches. For all SNP ranking methods the top 100 SNPs are presented, except for the outlier approach where 95 and 35 SNPs were identified as being under selection for dataset II and III, respectively.

Despite the differences described above, there was a substantial amount of overlap between the top SNPs among all ranking approaches (Fig. 6). For example, 59 to 68 of SNPs out of the top 100 were identified using paiwise *F*_ST_ and *Delta* in all three datasets. Similarly, large proportions of SNPs were shared between global *F*_ST_ and pairwise *F*_ST_ approach (51%–71%). In addition, a substantial proportion of outliers (up to 58%) was also ranked as top SNPs by the three other approaches (Fig. 6A).

In contrast to SNP ranking approaches, populations in the ascertainment group had a much larger effect on ranking of the most informative SNPs. For example, a relatively large proportion of SNPs (up to 80%) ranked by global *F*_ST_, pairwise *F*_ST_ and *Delta* was identified only in a single dataset (Fig. 6B) and a relatively large proportion of outliers was unique for each dataset (up to 50%).

### Performance of Top-ranked SNPs for GSI

**Figure 7:**
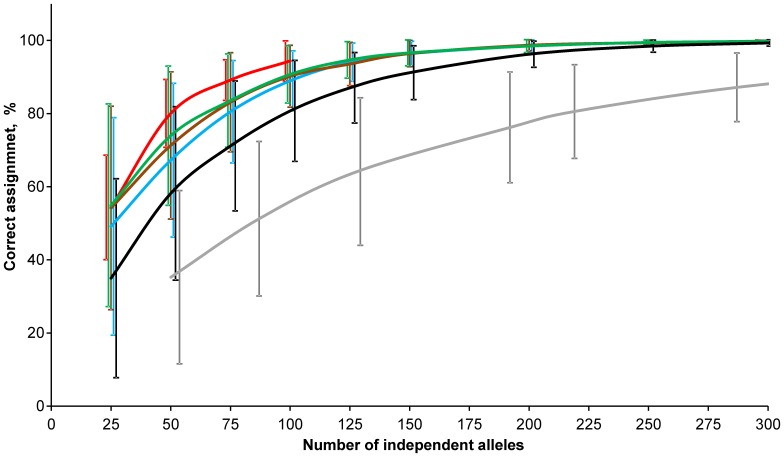
Overall assignment success for SNPs and STRs in dataset I. SNPs were ranked using i) global *F*_ST_ (blue), ii) pairwise *F*_ST_ (brown), iii) *Delta* (green) and iv) outlier approach (red). Overall assignment success of STRs and randomly chosen SNPs are shown as gray and black lines, respectively. The bars are representing standard deviation of assignment success among all populations for each ranking approach. The standard deviation bars were arranged for visual purposes to avoid overlapping.

Compared to randomly chosen sets of SNPs, the overall assignment success was considerably higher for top-ranked loci selected using four different approaches (Fig. 7). Of these, the outlier method identified the best performing loci, while loci selected using the global *F*_ST_ approach resulted in the lowest overall assignment accuracy. However, when the number of SNP markers reached 100, the overall assignment success for loci identified by global *F*_ST_, pairwise *F*_ST_ and *Delta* was rather similar, but still lower than for loci identified by the outlier method (Fig. 7, Table S4 in Appendix S1). In order to achieve 80%, 90% and 95% correct assignment for 23 Atlantic salmon populations, the outlier approach required 15–20% fewer loci than the three other approaches (Table 2). For example, 95% overall correct assignment was achieved using 94 outlier SNPs identified using population dataset II, whereas reaching the same level of assignment power with SNPs ranked by the three other approaches required 118 to 125 SNPs (Table 2).

**Table 2:** Estimated number of independent alleles of SNPs and STRs required to achieve 80%, 90%, and 95% overall correct assignment in 23 Atlantic salmon populations for each ranking method.

	Population dataset used for SNP ranking													
	I (23)				II (16)				III (6)					
	Global*F*_ST_	Pairwise*F*_ST_	*Delta*	Outlier	Global*F*_ST_	Pairwise*F*_ST_	*Delta*	Outlier	Global*F*_ST_	Pairwise*F*_ST_	*Delta*	Outlier	RandomSNPs	STRs*
80%	76	68	59	50	57	50	53	47	62	53	58	n/a	96	219 (∼18)
90%	113	104	100	81	90	82	87	71	98	87	95	n/a	149	336 (∼24)
95%	167	153	147	114	124	118	125	94	133	123	136	n/a	198	460 (∼28)

* Estimated number of STR loci is indicated in parentheses.

Although the overall GSI success was high, the number of SNPs required to provide similar accuracy varied in individual populations. For example, for Western Barents and Norwegian Sea populations, approximately 100–150 SNPs were necessary to attain >90% population assignment success, whereas 25–50 top SNPs were enough to attain similar level of correct assignment in the Eastern Barents, White and Baltic sea populations (Table S5 in Appendix S1). However, for markers ranked using population datasets II and III, i.e., when the most genetically distinct easternmost and Baltic populations were removed, the assignment success of >90% for Western Barents and Norwegian Sea salmon was achieved using 75–100 top-ranked SNPs (Table S5 in Appendix S1), except for a group of three populations with the lowest genetic divergence (pairwise *F*_ST_ over 2880 SNPs = 0.016–0.023; Table S3 in Appendix S1).

When comparing the assignment power for a given number of independent alleles between two marker classes, the performance of STRs was lower than that of random and top ranked SNPs. For example, an 80% overall assignment success was attained with 219 independent alleles for STRs (18 loci) while 96 and 47 independent alleles were sufficient to reach similar accuracy for random and top-ranked SNPs, respectively (Table 2, Fig. 7). On the other hand, when the assignment power was estimated for a given number of loci, STRs performed better than SNPs as less multi-allelic markers were needed to reach given level of assignment accuracy compared to bi-allelic markers. When evaluating the assignment success of individual populations, 18 STRs (219 independent alleles) were sufficient to assign salmon populations from the Baltic and the White and Eastern Barents seas with >99% accuracy, whereas all 31 STR markers (536 independent alleles) were required to achieve >90% accuracy for the Western Barents and Norwegian Sea populations. A combination of 31 STR markers and 25 top-ranked SNPs increased the overall assignment accuracy from 97% to 99% (Fig. S2 in Appendix S1).

### Validation of Top-ranked SNP on the Independent Dataset

The set of our top 100 SNPs identified in dataset II using the pairwise *F*_ST_ selection approach allowed >98% correct assignment in 13 out 26 European anadromous Atlantic salmon populations (Table S6 in Appendix S1). The lowest assignment accuracy was observed in British, Scottish and Irish populations (66%–87%) and was in line with the lower level of genetic differentiation among salmon populations in this area [33]. Similar to our North-European dataset, the number of top SNPs required to achieve 90% and 95% overall correct assignment for 26 European populations was considerably lower compared to randomly chosen SNPs (Table S7 in Appendix S1). However, the assignment accuracy of both top-ranked and random markers reached similar high levels (98%) when over 150 SNPs were used (Table S7 in Appendix S1).

**Figure 8:**
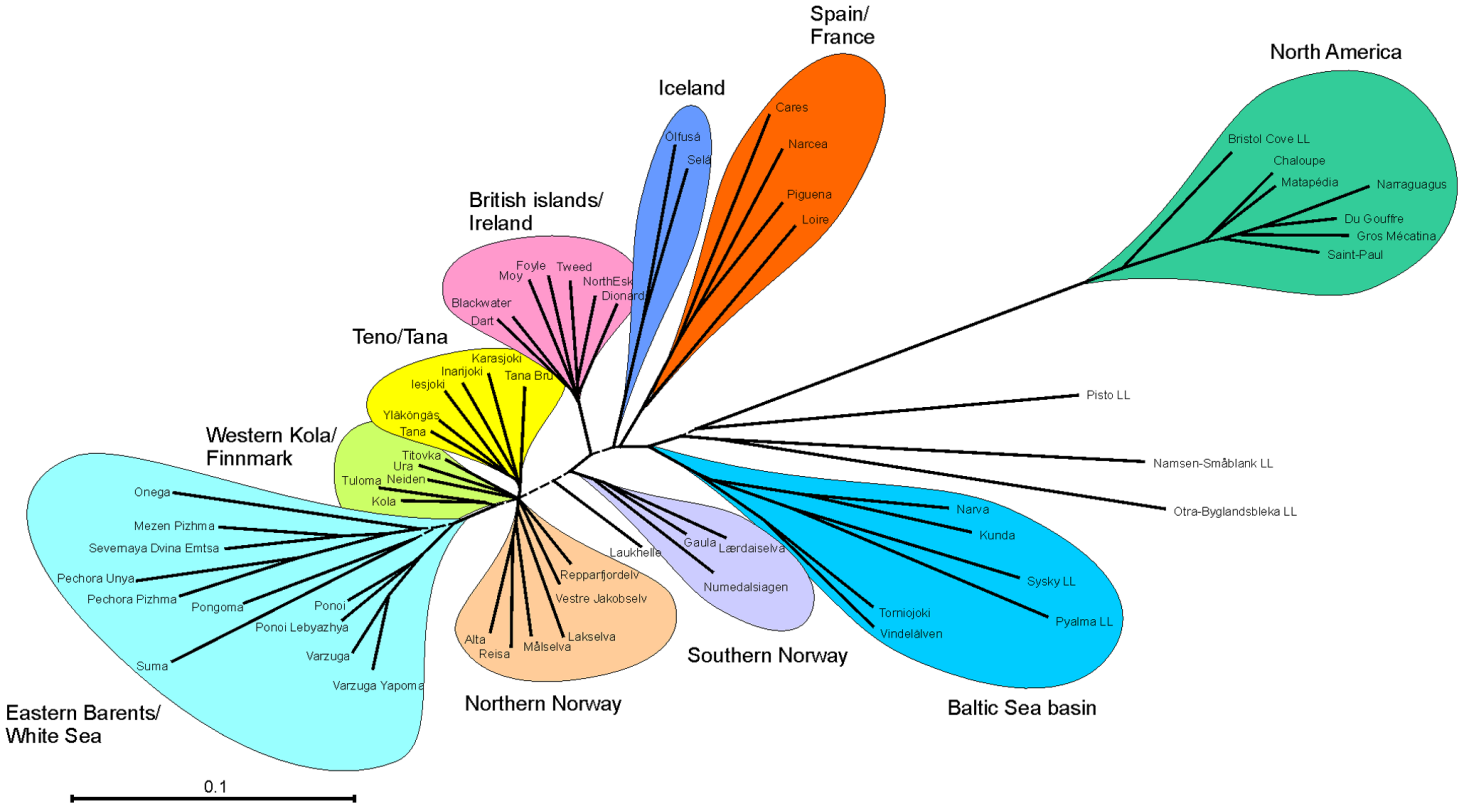
Genetic relationships among 61 Atlantic salmon populations throughout the species distribution range. Neighbour-joining dendrogram is based on Nei’s *D*_A_ genetic distances over 2763 SNPs. Landlocked populations are indicated with “LL”. The branches with bootstrap value support <80% are represented as dashed lines.

The constructed neighbor-joining tree consisting of 2763 SNPs from 61 population data derived by allelotyping and individual genotyping demonstrated the compatibility of the two approaches (Fig. 8): the genetic relationships of the populations were consistent with the results of Bourret et al. [20]. However, the combined dataset further revealed new insights into genetic relationships among populations such as separation between northern and southern Norwegian populations.

## Discussion

This is the first study that combines high-throughput SNP arrays and DNA pooling (i.e., allelotyping) to identify the most powerful sets of SNPs for genetic stock identification. We demonstrate how SNP arrays and DNA pooling enable a fast and cost-effective, yet reliable method for identifying the most informative markers among thousands of SNPs from large number of Atlantic salmon populations. In line with the previous studies of human populations [14], the results are most encouraging for projects involving high-sample throughput and low- to medium-multiplex SNP genotyping as a much smaller number of SNPs is needed for accurate genetic stock identification compared to randomly chosen SNPs. Moreover, we demonstrate the applicability of our approach on a data set compiled from two separate SNP genotyping projects and illustrate transferability of the data across studies without the need of laborious standardization in comparison with e.g. microsatellites [22].

### Reliability of DNA Pooling & Allelotyping

Recently, we showed that allelotyping of DNA pools is an effective method for reliable allele frequency estimation (individual genotyping *vs.* allelotyping, *Pearson’s r* = 0.992) [9]. To further validate the robustness of allelotyping, we compared our allele frequency estimates derived from allelotyping to allele frequencies obtained from individual genotyping by Bourret et al. [20] for three different river systems (Teno/Tana, Ponoi and Varzuga). Despite different individual samples, sampling locations and sampling years we observed very high correlation between the allele frequency estimates (*Pearson’s r* = 0.952–0.971) indicating the reliability of allelotyping approach. Moreover, the costs of analysis of DNA pools was about 15 times lower compared to the analysis of the same number of samples individually, i.e. the price for genome-wide analysis of DNA pools is at least an order of magnitude lower than individual genotyping [11]–[14].

This study also allowed a direct comparison of various population genetic parameters derived from allelotyping and genotyping of SNPs and STRs, respectively. We observed highly significant correlation between expected heterozygosity estimates for the two marker types. Similarly, the estimates of pairwise genetic differentiation demonstrated highly correlated patterns for SNP and STR markers. These results are in agreement with the earlier findings showing high concordance between different marker types [5], [6], [34], [35]. Likewise, the genetic relationships among populations inferred by SNPs and STRs were similar with high bootstrap clustering support for both marker classes. However, in contrast to other studies [20], [36], we observed a fine separation of the Western Barents salmon into two groups (Teno and Western Barents/Norwegian Sea). Furthermore, with a combined dataset consisting of 61 populations we were able to confirm the genetic relationships among populations over the whole distribution range as well as to reveal novel patterns such as clear separation between northern and southern Norwegian populations. Taken together, these results not only demonstrate the reliability of DNA pooling and allelotyping approach, but also illustrate one of the important advantages of SNPs – good transferability of the SNP data between independent studies. In contrast to STRs, which usually require laborious calibration and standardization of alleles [22], [37], this facilitates efficient compilation of large SNP datasets from independent studies creating further synergistic effects. However, SNP allele label switching may turn out problematic when SNP data sets from different genotyping platforms are compared.

### Detection and Performance of the most Informative SNPs

The evaluation of four SNP selection approaches demonstrated that all of them substantially improve the accuracy of GSI compared to randomly chosen SNPs. In most cases, SNPs selected using the outlier approach showed the highest assignment accuracy, whereas the global *F*_ST_ approach usually resulted in lower correct assignment rates. On the other hand, the differences in individual assignment success were evident only when the number of SNPs was below 100 while all SNP selection approaches enabled accurate GSI when more than 100 SNPs were used. These results are consistent with the earlier results demonstrating superior performance of outlier loci over neutral loci for genetic stock identification [30], [38], [39]. Similarly, the outperformance of pairwise SNP selection methods over the global *F*_ST_ approach has been shown in both humans [40] and cattle [8]. This is because the global *F*_ST_ approach tends to select for markers that are specific for the most distinct population or group of populations while the pairwise approaches allow selection of markers with high heterozygosity and more evenly distributed allele frequencies among populations, being thus more informative for individual assignment [8], [40], [41].

There was also a substantial amount of overlap between the top loci among all ranking approaches. For example, the overlap of top 100 SNPs selected using global *F*_ST_, pairwise *F*_ST_ or *Delta* ranged from 51 to 71%. Moreover, a large proportion (up to 58%) of the top 100 SNPs identified using global *F*_ST_, pairwise *F*_ST_ or *Delta* showed signs of divergent selection. This is consistent with the results of Lao et al. [7] showing that the five most informative SNPs dividing human populations from different continents exhibit the signs of local positive selection. Thus, our results indicate that despite the high assignment power of non-neutral markers, more simple pairwise methods are nearly as efficient in ranking the most informative loci for GSI.

On the other hand, our results indicate that the population dataset might play a larger role for identification of the most informative markers than the SNP selection approach [42], [43]. For example, the most informative set of loci selected using one set of human populations have been shown to lack power when applied to another set of populations [7], [42]. Indeed, our analysis revealed that the assignment accuracy was more affected by populations in the ascertainment group used for ranking SNPs rather than ranking method itself. While the SNPs ranked using population dataset I allowed quick discrimination of the populations at the large geographical scale, the markers ranked using population datasets II and III performed better at regional level. This can be explained by higher assignment success of the individuals from the Western Barents Sea and Norwegian Sea populations. Indeed, the geographically remote Baltic population and salmon of the White and Eastern Barents seas have very distinct genetic profiles that allow their discrimination using as few as 25–50 randomly chosen SNPs. On the other hand, genetic structure of salmon populations of the Western Barents and Norwegian seas is more subtle [18], [36] and GSI of these populations requires a higher number of markers. Thus, the exclusion of the easternmost salmon populations enabled detection of loci informative for the Western Barents Sea and Norwegian Sea salmon increasing the assignment accuracy of these populations. Given the results of our and previous [7] studies, pairwise-based methods for ranking the most informative markers for GSI are more robust to the ascertainment bias and thus are more applicable with extended datasets.

It must be recognized that our approach to perform power analyses of the top ranked SNPs for genetic stock identification yields overly optimistic accuracies, see [44]. Our accuracy levels are upwardly biased for two reasons: i) we simulated genotypes from estimated allele frequencies assuming the loci are independent of each other, and ii) we used the same set of samples to both select and evaluate the usefulness of the loci. To assure the accuracy of identified top SNPs for GSI we performed partial cross-validation of the loci on the completely independent dataset as has been proposed earlier [44]. Our 100 top ranked SNPs performed well for GSI among European anadromous Atlantic salmon, allowing >95% assignment success in 17 out of 26 populations. In comparison, 100 randomly chosen SNPs allowed the accuracy of >95% in 12 of 26 populations. Although the overall assignment accuracy of 100 top ranked SNPs (92%) was relatively similar to that of 100 randomly chosen SNPs (89%), the increase of GSI accuracy for particular populations (e.g. British and Irish) was up to 19% when using top ranked SNPs. Similar to our dataset, the assignment accuracy of both top-ranked and random markers reached comparable high levels when the number of SNPs exceeded 150 (95% and 96% for randomly chosen and top-ranked SNPs, respectively). These results indicate that our top-ranked loci can be efficiently applied for GSI in the whole European distribution range of Atlantic salmon.

### Assignment Power: SNPs vs. STRs

There are several ways to compare the effectiveness of different genetic marker classes for individual assignment: locus by locus, total number of alleles, and cost per information unit have been commonly applied [6], [45]. In this study 25 STR loci (374 independent alleles) provided similar GSI accuracy as ∼ 100 top-ranked SNP loci (100 independent alleles). This is consistent with recent comparisons between these two marker classes in chum (*Onchoryncus keta*) [45], sockeye (*Oncorhynchus nerka*) [46] and Atlantic salmon [6], demonstrating that more STR alleles are needed to achieve similar levels of assignment accuracy compared to the most informative SNPs identified from larger panels of markers.

The combination of 31 STR markers and 25 top-ranked SNPs increased the overall assignment success from 97% to 99%, representing a significant improvement of GSI by the reduction of the assignment error from 3% to 1%. Moreover, for some individual populations from the Western Barents and Norwegian seas, the combination of 31 STRs and 25 top-ranked SNPs allowed for an increase of assignment success up to 7%. This is in agreement with the results from studies on Chinook salmon (*Onchoryncus tschawytscha*), where a combination of 13 STRs and 92 SNPs provided a considerable increase of overall assignment success (from 76% to 84%) compared to application of both marker types separately [5]. Thus, addition of highly informative SNPs to already available STR panels may considerably increase the power of GSI even when the overall assignment accuracy of STRs is already high.

## Conclusions

This is the first study that demonstrates how the combination of SNP arrays and DNA pooling enables fast and cost-effective, yet reliable identification of the most informative markers among thousands of SNPs from large number of populations. The outlier approach was shown to be the most effective in ranking highly informative SNPs for GSI compared to three other methods (global *F*_ST_, pairwise *F*_ST_ and *Delta*). Compared to randomly chosen SNPs, the ranking procedures reduced the number of SNPs required for accurate GSI by up to 53%. However, GSI accuracy was more affected by populations in the ascertainment group rather than ranking method itself. We also demonstrated the usefulness of our top-SNPs on the independent set of populations covering nearly the whole European distribution range of Atlantic salmon. Taken together, this cost-effective approach described here for detection of the most informative SNPs for genetic stock identification can be readily adapted and applied for conservation and management of salmonids, as well as of other species.

## Supporting Information

Appendix S1 Contains the files: **Table S1.** Microsatellite loci sequencing details and genetic diversity indices: *A*_T_ – total number of alleles, *A*_R_ – allelic richness, *H*_O_ – observed heterozygosity, *H*_E_ – expected heterozygosity, *F*_ST_ – genetic differentiation. **Table S2.** Comparison of GSI accuracy (%) achieved using mixtures of fish with varying compositions in ONCOR [31], and using two alternative assignment approaches of Rannala & Mountain [32] and Paetkau et al. (1996). SNPs were ranked using pairwise *F*_ST_ selection method, dataset II. **Table S3.** Genetic distances (Reynolds et al. 1983) among 23 Atlantic salmon populations estimated on the basis of 2880 SNP (above diagonal) and 31 STR (below diagonal) markers. **Table S4.** Overall assignment success (%) for 50 and 100 top SNPs identified using different approaches depending on the population dataset. **Table S5.** GSI accuracy (%) in individual populations. **Table S6.** GSI accuracy (%) of SNPs ranked using pairwise *F*_ST_ (dataset II) tested on 26 independent individual populations (DRYAD entry doi:10.5061/dryad.gm367, Bourret et al. 2012). **Table S7.** Estimated number of SNPs required to achieve 80%, 90%, 95% and 98% overall correct assignments for independent dataset of 26 European Atlantic salmon populations. **Figure S1**. Histograms of the genetic differentiation estimates of each of 2880 SNPs, for each selection method and population dataset (x-axis scale is method-specific). The majority of the SNPs display low to moderate estimates of genetic differentiation and only a small proportion of SNPs display high levels of population differentiation. **Figure S2.** Overall assignment success with 31 STRs and additional top-ranked SNPs selected by global *F*_ST_ (dashed line), pairwise *F*_ST_ (solid line), *Delta* (long dash and dotted line) and outlier methods (dataset I).
(PDF)

Appendix S2 **Allele frequency estimates for 2880 SNPs and 23 populations used in the study.**
(XLSX)

Appendix S3 **The code of bespoken software used to convert allele frequencies into individual genotypes.**
(ZIP)

Appendix S4 **Genetic divergence indices per SNP locus estimated using 3 population datasets.**
(XLSX)
