# Supplementary material for: Finding Markers That Make a Difference: DNA Pooling and SNP-Arrays Identify Population Informative Markers for Genetic Stock Identification
Source: PLoS One. 2013 Dec 16;8(12):e82434. doi: 10.1371/journal.pone.0082434 (PMC3864958; doi:10.1371/journal.pone.0082434)
Supplement: Appendix S1 — Contains the files: Table S1. Microsatellite loci sequencing details and genetic diversity indices: A T – total number of alleles, A R – allelic richness, H O – observed heterozygosity, H E – expected heterozygosity, F ST – genetic differentiation. Table S2. Comparison of GSI accuracy (%) achieved using mixtures of fish with varying compositions in ONCOR [31], and using two alternative assignment approaches of Rannala & Mountain [32] and Paetkau et al. (1996). SNPs were ranked using pairwise F ST selection method, dataset II. Table S3. Genetic distances (Reynolds et al. 1983) among 23 Atlantic salmon populations estimated on the basis of 2880 SNP (above diagonal) and 31 STR (below diagonal) markers. Table S4. Overall assignment success (%) for 50 and 100 top SNPs identified using different approaches depending on the population dataset. Table S5. GSI accuracy (%) in individual populations. Table S6. GSI accuracy (%) of SNPs ranked using pairwise F ST (dataset II) tested on 26 independent individual populations (DRYAD entry doi:10.5061/dryad.gm367, Bourret et al. 2012). Table S7. Estimated number of SNPs required to achieve 80%, 90%, 95% and 98% overall correct assignments for independent dataset of 26 European Atlantic salmon populations. Figure S1. Histograms of the genetic differentiation estimates of each of 2880 SNPs, for each selection method and population dataset (x-axis scale is method-specific). The majority of the SNPs display low to moderate estimates of genetic differentiation and only a small proportion of SNPs display high levels of population differentiation. Figure S2. Overall assignment success with 31 STRs and additional top-ranked SNPs selected by global F ST (dashed line), pairwise F ST (solid line), Delta (long dash and dotted line) and outlier methods (dataset I). (PDF) [file pone.0082434.s001.pdf]

Appendix S1 for "**Finding Markers that Make a Difference: DNA Pooling and SNP-arrays Identify Population Informative Markers for Genetic Stock Identification**"

Mikhail Ozerov[1], Anti Vasemägi[2,8], Vidar Wennevik[3], Rogelio Diaz-Fernandez[1], Matthew Kent[4], John Gilbey[5], Sergey Prusov[6], Eero Niemelä[7], Juha-Pekka Vähä[1,*]

[1]Kevo Subarctic Research Institute, University of Turku, Turku, Finland
[2]Department of Biology, Division of Genetics and Physiology, University of Turku, Turku, Finland
[3]Research group Population Genetics and Ecology, Institute of Marine Research, Bergen, Norway
[4]Department of Animal and Aquacultural Sciences, Centre for Integrative Genetics (CIGENE), Norwegian University of Life Sciences, Ås, Norway
[5]Freshwater Laboratory, Marine Scotland, Faskally, Pitlochry, UK
[6]Freshwater Resources Laboratory, Knipovitch Polar Research Institute of Marine Fisheries and Oceanography, Murmansk, Russia
[7]Finnish Game and Fisheries Research Institute, Oulu, Finland
[8]Department of Aquaculture, Institute of Veterinary Medicine and Animal Science, Estonian University of Life Sciences, Tartu, Estonia

Table of contents:

Figure S1. Histograms of the genetic differentiation estimates of each of 2880 SNPs, for each selection method and population dataset (x-axis scale is method-specific). The majority of the SNPs display low to moderate estimates of genetic differentiation and only a small proportion of SNPs display high levels of population differentiation 2
Figure S2. Overall assignment success with 31 STRs and additional top-ranked SNPs selected by global F_{ST} (dashed line), pairwise F_{ST} (solid line), *Delta* (long dash and dotted line) and outlier methods (dataset I) 3
Table S1. Microsatellite loci sequencing details and genetic diversity indices: A_T – total number of alleles, A_R – allelic richness, H_O – observed heterozygosity, H_E – expected heterozygosity, F_{ST} – genetic differentiation 4
Table S2. Comparison of GSI accuracy (%) achieved using mixtures of fish with varying compositions in ONCOR [31], and using two alternative assignment approaches of Rannala & Mountain [32] and Paetkau et al. (1996). SNPs were ranked using pairwise F_{ST} selection method, dataset II 5
Table S3. Genetic distances (Reynolds et al. 1983) among 23 Atlantic salmon populations estimated on the basis of 2880 SNP (above diagonal) and 31 STR (below diagonal) markers 6
Table S4. Overall assignment success (%) for 50 and 100 top SNPs identified using different approaches depending on the population dataset 7
Table S5. GSI accuracy (%) in individual population 8
Table S6. GSI accuracy (%) of SNPs ranked using pairwise F_{ST} (dataset II) tested on 26 independent individual populations (DRYAD entry doi:10.5061/dryad.gm367, Bourret et al. 2012) 13
Table S7. Estimated number of SNPs required to achieve 80%, 90%, 95% and 98% overall correct assignments for independent dataset of 26 European Atlantic salmon populations 14

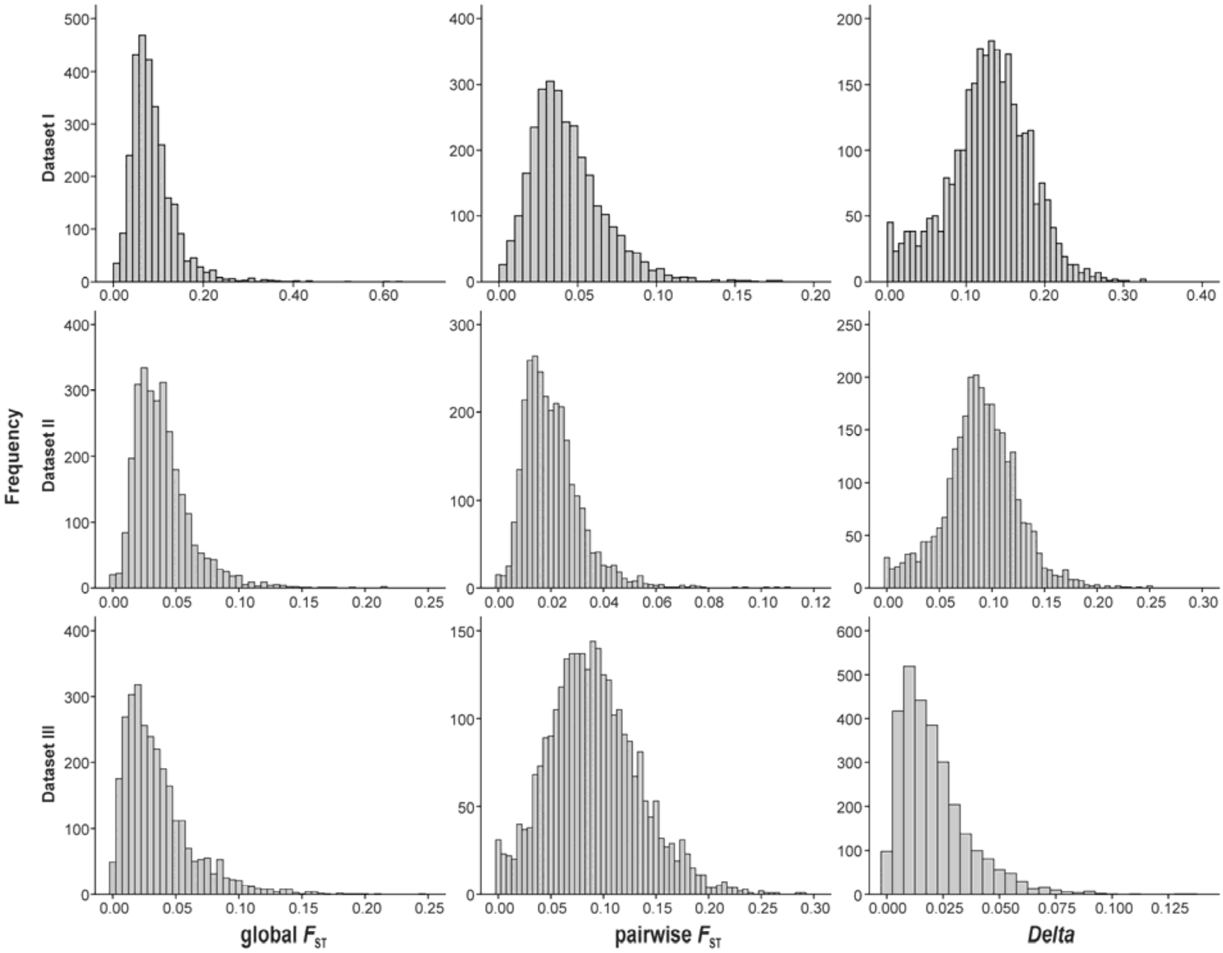

Figure S1. Histograms of the genetic differentiation estimates of each of 2880 SNPs, for each selection method and population dataset (x-axis scale is method-specific). The majority of the SNPs display low to moderate estimates of genetic differentiation and only a small proportion of SNPs display high levels of population differentiation.

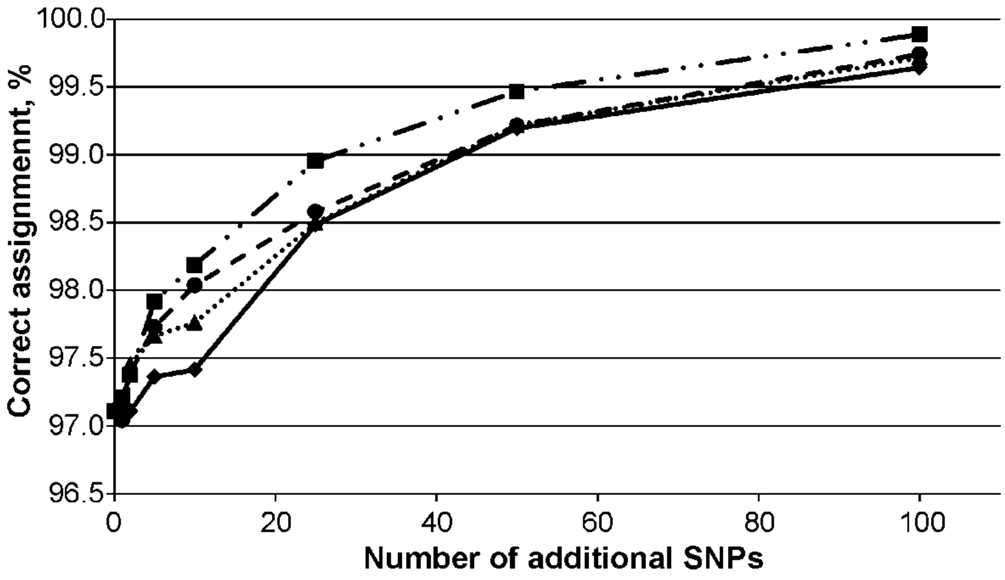

Figure S2. Overall assignment success with 31 STRs and additional top-ranked SNPs selected by global F_{ST} (dashed line), pairwise F_{ST} (solid line), *Delta* (long dash and dotted line) and outlier methods (dataset I).

Table S1. Microsatellite loci sequencing details and genetic diversity indices: A_T – total number of alleles, A_R – allelic richness, H_O – observed heterozygosity, H_E – expected heterozygosity, F_{ST} – genetic differentiation.

**Marker**	**Reference**		**Primer sequences**	A_T	A_R	H_O	H_E	F_{ST}
SSsp2215	Patterson et al. 2004	F:	GGTCAGTCAGTCACACCATGC	27	16.96	0.89	0.89	0.040
		R:	GTTTAGGTGTCCTGCCGGTCAAT					
Ssa171	O'Reilly et al. 1996	F:	ATTATCCAAAGGGGTCAAAA	27	12.20	0.82	0.81	0.055
		R:	GTTTGAGGTCGCTGGGGTTTACTAT					
SSsp2216	Patterson et al. 2004	F:	CTCCTCCTGGGATTTCCTGTCA	21	14.03	0.87	0.87	0.035
		R:	GTTTCTGGAGCAGAGGATTGCTG					
Ssa289	McConnell et al. 1995	F:	GTCATACAGTCACTATCATC	11	4.47	0.62	0.63	0.065
		R:	GTTTCTTTACAAATAGACAGACT					
Ssa14	McConnell et al. 1995	F:	CCTTTTGACAGATTTAGGATTTC	5	2.26	0.44	0.43	0.115
		R:	GTTTCAAACCAAACATACCTAAAGCC					
Ssa197	O'Reilly et al. 1996	F:	TGGCAGGGATTTGACATAAC	30	17.58	0.89	0.90	0.038
		R:	GGGTTGAGTAGGGAGGCTTG					
Ssa412	Cairney et al. 2000	F:	GTGGAGATACACAGCACTTA	9	3.77	0.54	0.54	0.144
		R:	GTTTCTTGGTTAGTACCGGACATG					
EST107	Vasemägi et al. 2005	F:	AGCGTTACGTCGAATCCAA	8	5.13	0.61	0.61	0.063
		R:	GTTTCTCATGGAGGGTGGAAGTGT					
SSf43	Sánchez et al. 1996	F:	GAGTCACTCAAAGTGAGGCC	12	4.73	0.34	0.34	0.045
		R:	GTTTAGCGGCATAACGTGCTGTGT					
Ssosl25	Slettan et al. 1995	F:	ATCTACACAGCTCCTGGTGGCAG	17	7.98	0.74	0.74	0.073
		R:	GTTTCATGTAATGGGTCGAGAGAAGTG					
Ssa98	O'Reilly et al. 1996	F:	GCAGTCCTTACCTGTGTGATTA	13	6.79	0.37	0.38	0.046
		R:	GTTTGGTAGTGATCTGGAGAGTGC					
Ssa202	O'Reilly et al. 1996	F:	TTCATGTGTTAATGTTGCGTG	16	10.65	0.85	0.83	0.052
		R:	GTTTCTTGGAATATCTAGAATATGGC					
SSA405	Cairney et al. 2000	F:	CTGAGTGGGAATGGACCAGACA	28	20.37	0.91	0.92	0.024
		R:	GTTTACTCGGGAGGCCCAGACTTGAT					
SSsp3016	AY372820	F:	GACAGGGCTAAGTCAGGTCA	19	12.88	0.77	0.78	0.063
		R:	GATTCTTATATACTCTTATCCCCAT					
SsaD486	King et al. 2005	F:	ACTCGGATAACACTCACAGGTC	5	1.37	0.01	0.01	0.025
		R:	GTT(T)CGCTGTGTATCAGTATTTTGG					
EST68	Vasemägi et al. 2005	F:	TGACACTGTGGCCTGTCTCT	8	6.31	0.59	0.61	0.070
		R:	GTTTGAGTTCTGGGTTATTTATTCACA					
MHCII	Stet et al. 2002	F:	GATGGCAAAGAGGAAAGTGAG	16	9.02	0.74	0.72	0.115
		R:	GTTTGTTATGCTCTACCTCTGAA					
MHC I	Grimholt et al. 2002	F:	___GAAGGTGCTGAAGAGGAACGTC	19	12.15	0.80	0.80	0.08
		R:	GTTTCAATTACCACAAGCCCGCTC					
Sleel53	U86704	F:	TGATTTGTTGCCTGCTGCTTCC	6	4.08	0.55	0.55	0.091
		R:	GTTTCCTGCTGCCCACATCATCC					
Ssleer15.1	U86708	F:	CATGTGCGTGTGCTTTTACAG	5	4.12	0.60	0.63	0.080
		R:	GTTTTCTGCATGTAGAACCCTGACC					
Sleen82	U86706	F:	CATGGAGAATCCCACTTTCTTA	11	7.10	0.72	0.72	0.065
		R:	GTTTCAGGGAGTGATATGGGACATAA					
SSsp2201	Patterson et al. 2004	F:	TTAGATGGTGGGATACTGGGAGGC	36	21.64	0.93	0.93	0.019
		R:	GTTTCGGGAGCCCCATAACCCTACTAATAAC					
SSspG7	Patterson et al. 2004	F:	CTTGGTCCCGTTCTTACGACAACC	22	14.28	0.83	0.82	0.046
		R:	GTTTGCACGCTGCTTGGTCCTTG					
SSsp1605	Patterson et al. 2004	F:	TCTGAGGCTCCTTCTACACTGA	11	7.47	0.77	0.76	0.067
		R:	GTTTGGTAGGTGCAAGAAAAAAGGAC					
EST19	Vasemägi et al. 2005	F:	CGCTTCCTGGACAAAAATTA	37	19.36	0.86	0.88	0.050
		R:	GTTTCATCTCTGTCATTCTCTTGC					
SSsp2210	Patterson et al. 2004	F:	CCTTTTTCCAATGGGATTCA	15	9.92	0.77	0.77	0.068
		R:	GTTTCATGCACACACATTCACTGC					
Ssosl85	Slettan et al. 1995	F:	TGTGGATTTTTGTATTATGTTA	21	11.05	0.77	0.78	0.059
		R:	GTTTATACATTTCCTCCTCATTCAG					
Ssa407	Cairney et al. 2000	F:	TCGTGACTACTAAGTCTTTGACCA	33	16.78	0.85	0.88	0.025
		R:	GTTTGTGTAGGCAGGTGTGGAC					
EST28	Vasemägi et al. 2005	F:	CACAGGCACACACTCCTCAT	13	4.49	0.33	0.32	0.039
		R:	GTTTCAGGTGAAGAGCATGACCAA					
SsaD144	King et al. 2005	F:	TCAATTGTTGGGTGCACATAG	35	24.11	0.93	0.93	0.029
		R:	GTTTGTGAAGGGGCTGACTAAC					
SsaD157	King et al. 2005	F:	GCTTAGGGCTGAGAGAGGAATAC	31	18.97	0.91	0.92	0.020
		R:	GTTTATCGAAATGGAACTTTTGAATG					

Table S2. Comparison of GSI accuracy (%) achieved using mixtures of fish with varying compositions in ONCOR [31], and using two alternative assignment approaches of Rannala & Mountain [32] and Paetkau et al. (1996). SNPs were ranked using pairwise F_{ST} selection method, dataset II.

	**Equally sized mixture**	**Mixture 1**	**Mixture 2**	**Mixture 3**	**Rannala & Mountain**	**Paetkau et al.**
25	0.59	0.58	0.59	0.56	0.55	0.55
50	0.82	0.80	0.80	0.80	0.82	0.83
75	0.89	0.88	0.89	0.90	0.90	0.91
100	0.94	0.94	0.94	0.93	0.94	0.95

Table S3. Genetic distances (Reynolds et al. 1983) among 23 Atlantic salmon populations estimated on the basis of 2880 SNP (above diagonal) and 31 STR (below diagonal) markers.

**Populations**	Laukhelle	Målselva	Reisa	Alta	Repparfjordelv	Lakselva	Iesjoki	Karasjoki	Inarijoki	Tana Bru	Yläkönäs	Vestre Jakobselv	Neiden	Titovka	Ura	Kola	Pechora Pizhma	Pechora Unya	Ponoi	Varzuga	Mezen Pizhma	Onega	Narva
Laukhelle	-	0.039	0.066	0.063	0.037	0.056	0.060	0.057	0.056	0.051	0.055	0.036	0.049	0.034	0.047	0.051	0.145	0.173	0.075	0.095	0.158	0.119	0.188
Målselva	0.035	-	0.033	0.035	0.033	0.046	0.046	0.057	0.053	0.047	0.040	0.034	0.050	0.040	0.051	0.054	0.146	0.173	0.084	0.106	0.159	0.121	0.185
Reisa	0.057	0.030	-	0.028	0.039	0.048	0.045	0.061	0.059	0.051	0.039	0.044	0.056	0.052	0.061	0.062	0.158	0.185	0.096	0.118	0.177	0.135	0.207
Alta	0.042	0.028	0.031	-	0.030	0.038	0.046	0.059	0.055	0.044	0.037	0.034	0.051	0.047	0.056	0.058	0.156	0.183	0.091	0.114	0.172	0.135	0.208
Repparfjordelv	0.030	0.020	0.031	0.022	-	0.032	0.036	0.033	0.029	0.024	0.028	0.020	0.029	0.022	0.030	0.035	0.142	0.168	0.062	0.084	0.152	0.117	0.192
Lakselva	0.048	0.036	0.040	0.026	0.030	-	0.060	0.059	0.055	0.046	0.051	0.036	0.052	0.045	0.056	0.059	0.163	0.191	0.088	0.111	0.175	0.140	0.215
Iesjoki	0.045	0.041	0.051	0.046	0.023	0.056	-	0.032	0.035	0.037	0.024	0.041	0.035	0.035	0.039	0.040	0.144	0.173	0.076	0.097	0.158	0.118	0.199
Karasjoki	0.051	0.056	0.061	0.056	0.034	0.067	0.023	-	0.030	0.034	0.033	0.039	0.033	0.032	0.038	0.040	0.153	0.181	0.071	0.095	0.167	0.130	0.209
Inarijoki	0.040	0.043	0.049	0.046	0.026	0.052	0.025	0.030	-	0.030	0.029	0.037	0.033	0.030	0.036	0.039	0.153	0.178	0.069	0.092	0.163	0.127	0.208
Tana Bru	0.056	0.051	0.059	0.050	0.028	0.054	0.035	0.040	0.036	-	0.023	0.031	0.029	0.027	0.035	0.039	0.151	0.177	0.068	0.091	0.156	0.126	0.204
Yläkönäs	0.040	0.038	0.048	0.040	0.019	0.044	0.025	0.033	0.024	0.017	-	0.034	0.032	0.031	0.039	0.040	0.144	0.171	0.075	0.097	0.156	0.120	0.195
Vestre Jakobselv	0.038	0.031	0.040	0.023	0.022	0.033	0.040	0.051	0.040	0.044	0.034	-	0.033	0.025	0.033	0.038	0.141	0.167	0.065	0.086	0.153	0.115	0.194
Neiden	0.043	0.043	0.049	0.036	0.028	0.051	0.032	0.037	0.030	0.037	0.031	0.031	-	0.018	0.023	0.029	0.142	0.170	0.058	0.081	0.151	0.116	0.199
Titovka	0.030	0.031	0.044	0.032	0.022	0.041	0.026	0.033	0.026	0.037	0.026	0.026	0.020	-	0.016	0.019	0.125	0.153	0.040	0.060	0.133	0.100	0.183
Ura	0.039	0.040	0.049	0.037	0.027	0.048	0.030	0.037	0.031	0.038	0.029	0.029	0.022	0.017	-	0.025	0.134	0.162	0.046	0.066	0.142	0.108	0.194
Kola	0.042	0.042	0.054	0.045	0.033	0.052	0.030	0.040	0.035	0.044	0.038	0.040	0.033	0.019	0.025	-	0.126	0.155	0.037	0.054	0.131	0.097	0.192
Pechora Pizhma	0.113	0.116	0.122	0.104	0.098	0.118	0.106	0.110	0.114	0.108	0.105	0.103	0.092	0.097	0.096	0.094	-	0.079	0.133	0.149	0.198	0.102	0.276
Pechora Unya	0.116	0.120	0.130	0.113	0.104	0.125	0.118	0.123	0.124	0.120	0.114	0.111	0.107	0.106	0.106	0.101	0.070	-	0.163	0.179	0.235	0.142	0.303
Ponoi	0.060	0.054	0.064	0.060	0.044	0.063	0.042	0.054	0.048	0.048	0.042	0.048	0.044	0.030	0.034	0.031	0.096	0.095	-	0.026	0.130	0.102	0.219
Varzuga	0.060	0.067	0.073	0.064	0.057	0.068	0.051	0.060	0.053	0.068	0.057	0.058	0.055	0.035	0.046	0.032	0.108	0.109	0.026	-	0.144	0.117	0.238
Mezen Pizhma	0.093	0.084	0.095	0.076	0.075	0.094	0.077	0.088	0.083	0.081	0.074	0.076	0.067	0.062	0.070	0.066	0.078	0.102	0.062	0.082	-	0.140	0.290
Onega	0.115	0.110	0.123	0.124	0.103	0.130	0.096	0.114	0.111	0.111	0.105	0.106	0.101	0.082	0.086	0.077	0.143	0.151	0.069	0.087	0.097	-	0.243
Narva	0.123	0.123	0.134	0.118	0.116	0.125	0.129	0.137	0.129	0.131	0.123	0.124	0.125	0.110	0.121	0.117	0.189	0.200	0.131	0.141	0.166	0.183	-

Table S4. Overall assignment success (%) for 50 and 100 top SNPs identified using different approaches depending on the population dataset.

	Ranking approach				
100	global F_{ST}	pairwise F_{ST}	*Delta*	Outlier	Random*
Dataset I	88.97	90.21	90.71	94.40	80.12 (±1.02)
Dataset II	94.15	94.40	93.68	95.13	80.12 (±1.02)
Dataset III	92.69	94.24	92.17	n/a	80.12 (±1.02)
50					
Dataset I	67.26	71.29	73.97	80.05	59.43 (±2.27)
Dataset II	77.48	81.53	79.54	79.94	59.43 (±2.27)
Dataset III	75.10	78.64	75.35	n/a	59.43 (±2.27)

*Averaged across 5 tests (standard deviation is shown in brackets)

Table S5. GSI accuracy (%) in individual populations.

Population	No of SNPs	Population dataset I				Population dataset II				Population dataset III				Random SNPs
		global F_{ST}	pairwise F_{ST}	*Delta*	outlier	global F_{ST}	pairwise F_{ST}	*Delta*	outlier	global F_{ST}	pairwise F_{ST}	*Delta*	outlier	
Laukhelle	25	72.40	68.40	59.09	69.41	88.92	79.55	77.17	79.39	94.74	91.39	83.67	85.35	37.22
	50	82.69	82.48	86.27	92.82	99.18	96.13	94.83	93.36	98.49	97.86	95.70		69.67
	75	94.42	93.40	90.95	97.12	99.41	98.40	98.39	97.86	99.91	98.83	98.26		84.86
	100	96.27	97.63	94.85	98.47	99.78	99.91	99.77		99.99	99.97	99.49		81.85
	125	98.79	99.12	98.27		100.00	99.92	99.95		100.00	99.97	99.94		94.10
	150	99.62	99.30	99.59		99.96	99.98	99.85		100.00	100.00	99.99		96.99
	200	99.96	99.70	99.78		100.00	100.00	99.99		100.00	100.00	99.99		99.87
	250	100.00	99.99	99.77		100.00	100.00	100.00		100.00	100.00	100.00		99.57
	300	99.99	100.00	100.00		100.00	100.00	100.00		100.00	100.00	100.00		99.90
Målselva	25	35.08	37.81	50.87	55.99	72.74	66.11	63.33	59.68	69.19	65.56	52.58	62.96	24.42
	50	62.73	72.40	76.67	83.85	93.04	89.90	87.43	88.74	90.93	89.74	81.42		50.65
	75	80.16	89.58	86.11	95.60	98.92	96.34	95.32	95.38	94.61	97.47	92.42		66.73
	100	91.26	96.47	95.34	98.14	99.20	98.55	98.55		99.10	98.19	97.28		76.94
	125	97.33	97.41	95.69		99.93	99.82	99.45		99.21	99.63	97.55		83.94
	150	99.31	99.45	98.89		99.86	99.85	99.39		99.81	99.43	99.15		92.82
	200	99.97	99.37	99.60		99.99	99.98	99.57		99.88	99.72	99.87		97.08
	250	99.80	99.50	99.99		100.00	100.00	100.00		100.00	99.82	99.70		99.24
	300	100.00	100.00	99.96		100.00	100.00	100.00		100.00	100.00	99.94		99.83
Reisa	25	48.27	57.12	60.47	52.48	60.94	57.64	59.90	63.47	60.67	80.20	79.77	59.80	20.08
	50	66.37	71.45	78.35	78.38	84.20	85.66	80.40	80.59	91.18	94.32	93.30		64.15
	75	78.69	84.77	86.89	90.23	94.64	94.28	91.68	92.17	95.06	98.23	97.25		75.19
	100	89.31	94.93	93.28	96.15	98.10	98.11	97.12		98.68	99.36	97.84		87.31
	125	96.70	97.26	96.71		99.29	98.82	99.30		99.36	99.62	98.85		90.70
	150	96.84	97.74	98.13		99.38	99.60	99.14		99.38	99.86	99.82		92.27
	200	99.31	99.42	99.11		99.60	99.99	99.72		100.00	99.94	99.81		96.16
	250	99.27	99.61	99.73		100.00	100.00	99.97		99.99	100.00	100.00		99.24
	300	99.83	99.99	99.76		99.99	99.98	99.99		99.99	100.00	99.99		99.63
Alta	25	35.64	44.81	46.40	60.88	61.28	71.21	71.88	67.04	57.64	69.91	62.26	64.88	25.58
	50	62.70	71.84	75.04	77.18	85.79	88.91	87.67	81.61	83.67	86.32	87.56		48.78
	75	74.63	80.61	85.68	91.14	94.85	95.42	93.95	91.33	91.59	96.53	96.17		69.52
	100	86.44	94.89	92.63	94.42	97.57	97.45	97.44		96.49	97.74	95.95		81.55
	125	95.07	96.08	94.59		98.78	99.09	98.77		98.28	99.32	97.61		86.53
	150	97.71	97.69	97.94		99.38	99.52	99.16		99.27	99.65	99.13		89.31
	200	98.61	99.24	98.85		99.80	99.89	99.79		99.66	99.68	99.94		97.17
	250	99.63	99.66	99.42		100.00	99.99	100.00		99.68	99.96	99.92		98.93
	300	99.91	99.99	99.77		100.00	99.99	99.99		100.00	99.95	99.99		99.80
Repparfjordelv	25	22.09	29.22	31.70	39.72	38.75	53.57	37.88	50.85	31.74	34.53	30.44	47.62	16.57
	50	44.93	52.26	53.81	73.19	66.70	69.40	71.19	77.62	60.16	64.79	58.63		33.47
	75	60.53	70.00	68.48	85.17	84.94	81.57	80.22	90.77	77.46	82.59	80.71		45.34
	100	78.53	81.55	83.21	91.24	91.76	91.40	88.83		84.54	89.87	85.45		66.90
	125	86.21	85.44	89.81		94.54	94.65	93.39		91.51	92.45	93.56		71.01
	150	91.72	92.78	93.61		97.80	97.57	95.97		95.79	95.14	96.18		83.30
	200	94.60	96.88	96.03		99.06	99.61	98.38		98.09	98.72	98.60		90.36
	250	98.63	98.67	98.44		99.88	99.83	98.96		99.14	99.54	99.11		95.15
	300	99.57	99.57	99.50		99.98	99.97	99.54		99.94	99.83	99.10		98.21
Lakselva	25	31.83	46.59	49.87	59.37	71.71	66.17	60.52	66.92	31.65	59.38	43.49	59.20	18.79
	50	62.46	73.73	81.61	87.04	86.37	88.37	81.26	88.63	78.89	84.16	75.75		63.41
	75	83.20	90.56	87.99	93.77	96.47	96.99	95.01	95.86	86.29	91.82	90.62		68.38
	100	95.56	95.46	94.92	96.69	98.68	99.12	97.61		93.86	95.97	93.73		86.68
	125	97.90	96.59	98.30		99.30	99.74	98.76		97.45	98.12	95.05		87.84
	150	99.03	98.23	98.69		99.82	99.74	99.53		99.06	98.86	98.41		93.74
	200	99.89	99.55	99.75		100.00	100.00	99.91		99.71	99.67	99.40		97.65
	250	99.63	99.87	99.99		99.99	100.00	100.00		99.95	99.74	99.98		99.75
	300	100.00	100.00	99.87		100.00	100.00	99.94		99.97	100.00	99.93		99.98
Iesjoki	25	28.67	31.90	23.36	53.35	38.18	43.92	46.14	55.61	24.93	34.41	26.47	43.19	19.63
	50	55.27	49.47	54.93	79.10	77.10	85.74	84.21	77.87	62.60	69.43	59.35		41.35
	75	74.56	70.59	74.15	91.40	89.64	94.25	91.87	91.30	83.00	88.05	82.97		69.43
	100	82.87	80.90	85.19	95.50	97.91	97.47	95.64		91.79	96.57	90.77		79.71
	125	89.49	93.59	95.32		97.94	99.01	98.02		94.70	97.24	95.91		88.10
	150	95.30	96.34	97.13		99.68	99.59	98.95		97.01	99.31	97.47		91.45
	200	98.51	99.19	99.27		99.98	99.99	99.84		99.60	99.72	99.30		96.52
	250	98.86	99.77	99.44		99.91	99.99	99.90		99.79	99.95	99.73		98.34
	300	99.96	99.84	99.98		100.00	100.00	99.91		99.77	100.00	99.93		98.92

Table S5. GSI accuracy (%) in individual populations (cont.).

**Population**	**No of SNPs**	**Population dataset I**				**Population dataset II**				**Population dataset III**				**Random SNPs**
		global F_{ST}	pairwise F_{ST}	*Delta*	Outlier	global F_{ST}	pairwise F_{ST}	*Delta*	Outlier	global F_{ST}	pairwise F_{ST}	*Delta*	Outlier	
Karasjoki	25	32.45	38.94	45.95	34.50	31.56	34.07	33.04	30.76	25.42	35.50	32.01	27.08	21.48
	50	52.11	54.23	62.58	58.55	65.44	70.75	71.43	64.66	54.73	57.13	55.50		50.61
	75	65.46	74.77	77.59	76.87	79.30	84.64	83.13	86.00	68.17	80.48	73.28		62.58
	100	81.81	82.29	90.64	89.91	92.88	92.66	94.01		85.16	87.88	86.22		77.65
	125	91.06	89.42	95.05		96.53	95.94	97.01		92.23	91.71	88.73		84.24
	150	96.48	94.37	96.24		97.80	98.70	97.83		96.31	96.60	95.13		93.72
	200	97.50	98.46	98.73		99.72	99.44	99.66		98.01	98.44	97.79		97.02
	250	99.20	99.00	99.90		99.90	99.82	99.91		99.23	99.21	99.50		98.79
	300	99.81	99.46	99.74		99.99	100.00	99.99		99.81	99.81	99.26		99.47
Inarijoki	25	25.42	28.55	31.37	35.43	30.23	30.90	31.37	34.70	23.32	30.73	33.07	29.08	13.72
	50	51.80	57.57	54.10	69.05	52.80	72.77	66.39	66.06	54.71	62.87	64.12		29.79
	75	73.17	78.53	76.49	77.10	78.65	88.06	82.46	89.26	75.61	81.63	76.09		64.33
	100	85.19	84.83	85.32	92.31	93.81	94.05	93.99		84.24	89.27	84.88		70.35
	125	93.43	87.53	91.88		95.81	96.03	95.70		90.51	92.45	89.93		83.73
	150	95.66	96.17	92.62		98.55	98.71	98.53		95.03	97.47	94.07		84.16
	200	98.12	98.01	98.37		98.95	99.71	99.56		98.24	98.56	97.83		93.26
	250	99.53	99.33	99.27		99.98	99.73	99.91		99.29	99.10	98.86		98.99
	300	99.83	99.86	99.82		100.00	99.97	100.00		99.92	99.95	99.53		99.59
Tana Bru	25	22.57	29.84	25.40	31.71	26.51	30.59	38.20	44.76	23.53	28.60	34.68	34.37	14.88
	50	44.33	55.49	50.50	68.29	68.25	73.14	68.01	64.21	45.61	53.85	53.16		45.18
	75	60.98	68.34	69.59	83.16	82.34	82.92	80.71	85.98	73.63	77.84	75.54		56.14
	100	80.37	79.44	85.28	91.14	91.81	91.75	87.61		84.80	86.87	88.80		69.38
	125	83.82	83.60	91.95		93.60	95.83	92.25		87.82	92.68	91.59		78.14
	150	91.46	88.14	93.21		98.02	97.07	97.49		93.27	95.86	95.04		87.05
	200	96.98	94.76	97.32		99.58	99.08	98.37		98.18	98.15	99.16		93.91
	250	99.21	98.94	99.11		99.35	99.68	99.43		99.30	99.40	99.43		96.71
	300	99.76	99.51	99.83		100.00	99.99	99.85		99.79	99.87	99.83		99.34
Yläköngäs	25	28.86	35.60	36.74	47.45	50.08	51.61	40.85	64.66	31.65	44.95	39.21	60.44	19.55
	50	47.24	53.49	60.98	79.00	71.95	83.44	73.41	79.18	72.50	74.88	66.30		34.03
	75	69.26	72.07	67.49	88.24	81.99	92.25	83.90	90.94	85.73	88.53	77.44		63.46
	100	80.87	79.70	84.76	95.78	94.86	95.94	94.10		90.71	96.16	89.43		72.52
	125	91.81	87.84	89.42		95.10	97.68	97.05		94.30	96.32	96.00		74.10
	150	93.14	92.29	94.19		98.83	98.91	96.94		96.33	97.68	96.08		79.30
	200	98.02	98.25	97.59		99.13	99.25	99.75		99.22	99.57	99.23		93.29
	250	99.32	98.94	99.50		99.89	99.95	99.64		99.29	99.86	99.64		97.09
	300	99.42	99.81	99.73		100.00	99.99	99.85		99.88	99.99	99.82		98.85
Vestre Jakobselv	25	22.18	28.53	49.67	42.64	58.86	46.01	49.67	53.40	45.79	44.03	33.17	52.77	24.79
	50	51.50	54.37	81.94	81.83	84.75	81.50	81.94	81.99	67.28	74.66	60.63		36.98
	75	71.82	74.29	89.50	86.93	90.16	86.95	89.50	92.77	79.12	83.75	83.57		52.55
	100	78.75	85.45	92.84	93.94	94.87	92.78	92.84		88.94	91.91	94.22		69.65
	125	92.04	86.49	97.22		97.12	96.92	97.22		94.08	94.40	92.31		78.51
	150	91.23	95.25	98.80		98.80	97.90	98.80		97.06	95.94	97.17		85.92
	200	96.72	97.08	99.45		99.80	99.26	99.45		99.02	99.12	98.67		96.05
	250	99.07	98.20	99.97		99.85	99.13	99.97		99.71	99.69	99.49		97.64
	300	99.96	99.56	99.87		99.89	99.67	99.87		99.97	99.98	99.68		99.24
Neiden	25	24.36	26.69	18.20	26.52	34.09	35.39	18.20	28.51	33.11	39.17	45.08	40.58	17.20
	50	43.93	50.95	50.51	58.54	54.90	54.00	50.51	55.11	69.61	71.68	59.11		46.26
	75	66.49	58.80	75.90	70.31	67.57	70.69	75.90	80.38	81.68	84.18	77.61		54.69
	100	80.72	79.62	84.69	82.60	84.47	82.87	84.69		89.18	91.66	87.92		69.31
	125	86.45	86.76	92.26		90.45	88.39	92.26		94.35	95.40	91.04		77.50
	150	93.49	90.86	96.15		93.23	92.97	96.15		97.19	96.43	95.79		84.04
	200	96.69	97.83	98.33		98.71	97.15	98.33		98.66	98.89	97.97		95.63
	250	98.89	98.48	99.54		99.50	98.72	99.54		99.84	99.33	98.86		97.42
	300	99.20	99.64	99.97		99.91	99.89	99.97		99.98	99.90	99.87		98.59
Titovka	25	24.43	31.50	22.26	30.36	26.11	25.06	22.26	33.12	22.13	32.59	27.25	29.11	13.98
	50	48.07	45.57	51.64	50.68	54.58	42.34	51.64	66.64	55.75	50.17	45.11		30.34
	75	64.92	61.59	70.91	71.05	68.71	63.01	70.91	77.38	67.83	72.13	62.34		39.49
	100	79.35	77.43	80.55	80.25	73.40	74.33	80.55		82.09	84.62	75.83		52.99
	125	86.19	85.17	87.36		89.92	82.60	87.36		87.95	87.28	81.45		69.38
	150	90.13	90.48	95.21		93.20	89.95	95.21		92.62	91.63	90.68		78.30
	200	94.90	95.63	97.24		96.93	96.65	97.24		95.77	96.13	95.11		86.31
	250	98.26	97.22	98.67		98.18	96.88	98.67		98.87	98.68	98.05		94.72
	300	99.46	99.20	99.61		99.48	98.44	99.61		99.59	99.67	98.42		96.78

Table S5. GSI accuracy (%) in individual populations (cont.).

**Population**	**No of SNPs**	**Population dataset I**				**Population dataset II**				**Population dataset III**				**Random SNPs**
		global F_{ST}	pairwise F_{ST}	*Delta*	Outlier	global F_{ST}	pairwise F_{ST}	*Delta*		global F_{ST}	pairwise F_{ST}	*Delta*		
Ura	25	25.24	37.96	41.82	32.96	27.88	34.07	36.69	23.12	34.17	36.09	39.70	30.96	14.66
	50	48.21	54.80	58.69	62.75	52.84	57.06	57.42	57.56	63.70	59.72	60.20		37.87
	75	70.40	71.46	74.44	77.60	70.03	69.50	68.14	81.77	82.65	77.65	71.87		56.92
	100	81.66	86.47	81.49	86.65	78.14	81.29	83.25		87.06	87.60	81.02		62.32
	125	89.49	89.35	88.05		89.98	88.21	86.41		93.08	93.47	88.34		82.68
	150	94.59	93.74	91.76		94.08	93.34	95.09		95.47	96.93	93.97		82.78
	200	97.94	97.50	96.36		97.40	96.52	96.70		98.32	98.57	97.29		93.44
	250	98.33	98.95	99.05		99.34	99.38	97.84		99.51	99.09	98.40		96.11
	300	99.28	99.59	98.99		99.78	99.56	99.72		99.88	99.96	99.35		97.77
Kola	25	33.06	28.55	40.09	47.86	35.45	50.44	48.09	45.61	24.14	31.25	34.26	26.95	18.27
	50	58.40	55.00	63.85	70.88	59.95	67.98	66.62	69.41	50.62	66.42	61.51		42.05
	75	76.99	79.09	77.08	88.65	84.40	82.27	79.90	86.01	76.05	81.04	74.68		61.22
	100	87.06	83.93	86.35	91.82	90.64	93.76	91.06		88.14	89.44	82.72		69.96
	125	92.32	92.87	93.29		96.14	96.12	93.35		92.67	93.99	91.33		83.89
	150	94.19	95.77	97.12		97.87	97.64	96.86		94.56	95.12	93.48		85.38
	200	98.40	99.07	98.15		99.61	98.59	98.86		97.93	98.42	96.30		93.69
	250	99.61	99.40	99.41		99.88	99.79	99.39		98.94	99.44	98.58		96.86
	300	99.59	99.46	99.67		99.90	99.82	99.95		99.77	99.83	99.03		98.59
Ponoi	25	57.94	72.03	76.33	60.87	41.43	48.35	52.75	47.66	35.96	45.40	40.19	40.02	28.35
	50	81.06	90.75	92.31	91.16	64.24	76.40	79.64	74.89	65.76	76.20	78.81		60.62
	75	92.18	95.95	96.11	95.22	81.14	84.30	91.12	87.54	89.96	90.47	88.29		72.01
	100	94.14	97.10	98.22	98.21	89.99	92.16	92.62		93.99	91.50	92.84		88.10
	125	97.13	98.52	98.83		93.22	95.84	95.65		93.35	95.17	97.03		92.93
	150	98.55	99.32	99.67		96.37	97.34	97.56		96.06	96.88	98.77		97.75
	200	99.33	99.95	99.63		98.92	98.89	99.20		98.53	98.87	99.65		98.15
	250	99.77	99.81	99.98		99.10	99.58	99.76		99.48	99.57	99.96		99.61
	300	99.98	100.00	100.00		99.84	99.56	99.98		100.00	99.87	99.98		99.85
Varzuga	25	71.99	87.86	89.26	69.94	44.86	71.91	65.56	51.20	41.90	67.57	72.36	51.72	49.28
	50	84.67	94.31	91.50	92.31	86.34	82.27	86.62	85.62	81.65	85.91	85.77		73.15
	75	93.89	96.76	97.79	96.47	87.97	90.66	93.70	90.99	91.10	95.33	92.45		83.73
	100	96.40	96.88	98.31	98.45	93.23	95.15	93.46		93.70	93.65	95.62		95.61
	125	98.04	98.67	99.28		94.56	98.12	97.09		95.21	97.50	98.06		95.09
	150	98.90	99.37	99.52		96.81	98.24	98.22		97.46	98.33	98.87		98.96
	200	99.74	99.79	99.85		98.61	99.40	99.34		98.59	98.65	99.62		97.90
	250	99.78	99.98	99.55		99.24	99.92	99.57		99.84	99.54	99.97		99.39
	300	99.96	99.94	99.99		99.96	99.73	99.65		99.79	99.78	100.00		99.76
Pechora Pizhma	25	96.11	91.92	92.04	55.90	67.58	73.29	82.59	67.55	73.26	72.76	72.85	62.16	80.68
	50	99.48	99.73	99.45	94.28	92.56	96.58	97.91	91.84	90.75	94.82	95.24		92.34
	75	99.99	100.00	99.78	97.49	99.11	99.24	99.46	97.69	97.68	99.03	99.65		96.15
	100	99.89	99.99	100.00	99.74	99.44	99.98	99.95		99.59	99.66	99.87		98.83
	125	100.00	100.00	100.00		99.99	99.99	99.99		99.99	99.79	99.99		99.77
	150	100.00	100.00	100.00		99.99	100.00	100.00		99.98	99.97	100.00		99.95
	200	100.00	100.00	100.00		100.00	100.00	99.96		100.00	100.00	100.00		100.00
	250	100.00	100.00	100.00		100.00	100.00	100.00		100.00	100.00	100.00		100.00
	300	100.00	100.00	100.00		100.00	100.00	100.00		100.00	100.00	100.00		100.00
Pechora Unya	25	97.86	98.96	96.98	77.32	78.41	85.27	89.68	87.03	84.63	83.46	83.18	79.12	76.46
	50	99.62	99.79	99.85	98.94	97.04	97.87	97.98	97.36	95.59	97.43	97.99		95.25
	75	99.99	99.99	99.92	99.37	99.26	99.92	99.69	98.82	97.63	98.62	99.89		98.25
	100	99.96	100.00	99.98	99.98	99.67	99.76	99.98		99.86	99.53	99.92		99.54
	125	100.00	100.00	99.95		99.94	99.93	99.79		99.99	99.92	99.99		99.88
	150	100.00	100.00	100.00		100.00	100.00	100.00		100.00	100.00	100.00		100.00
	200	100.00	100.00	100.00		100.00	100.00	100.00		100.00	100.00	99.99		100.00
	250	100.00	100.00	100.00		100.00	100.00	100.00		100.00	100.00	100.00		100.00
	300	100.00	100.00	100.00		100.00	100.00	100.00		100.00	100.00	100.00		100.00
Mezen Pizhma	25	95.31	93.76	92.94	76.47	80.37	91.64	90.82	72.67	77.68	81.24	80.02	65.46	65.60
	50	99.42	99.98	99.74	94.67	98.37	98.97	99.78	96.83	94.78	96.90	97.97		93.64
	75	100.00	99.99	99.97	98.33	99.97	99.95	99.87	98.94	99.05	99.31	99.73		95.39
	100	100.00	99.99	100.00	99.79	99.97	100.00	99.79		99.89	99.99	99.99		99.63
	125	100.00	100.00	100.00		100.00	100.00	100.00		100.00	99.89	100.00		99.88
	150	100.00	100.00	100.00		100.00	100.00	100.00		100.00	100.00	100.00		99.98
	200	100.00	100.00	100.00		100.00	100.00	100.00		100.00	100.00	100.00		100.00
	250	100.00	100.00	100.00		100.00	100.00	100.00		100.00	100.00	100.00		100.00
	300	100.00	100.00	100.00		100.00	100.00	100.00		100.00	100.00	100.00		100.00

Table S5. GSI accuracy (%) in individual populations (cont.).

**Population**	**No of SNPs**	**Population dataset I**				**Population dataset II**				**Population dataset III**				**Random SNPs**
		global F_{ST}	pairwise F_{ST}	*Delta*	Outlier	global F_{ST}	pairwise F_{ST}	*Delta*	Outlier	global F_{ST}	pairwise F_{ST}	*Delta*	Outlier	
Onega	25	98.63	99.56	98.88	89.12	81.08	93.64	89.62	89.06	78.49	94.26	96.92	91.88	84.53
	50	100.00	100.00	99.99	98.57	95.78	99.53	99.47	98.85	98.40	99.53	99.95		98.38
	75	100.00	100.00	100.00	99.63	99.98	100.00	100.00	99.82	99.91	99.99	100.00		99.86
	100	100.00	100.00	100.00	100.00	100.00	100.00	100.00		99.99	100.00	100.00		99.99
	125	100.00	100.00	100.00		100.00	100.00	100.00		100.00	100.00	99.99		100.00
	150	100.00	100.00	100.00		100.00	100.00	100.00		100.00	100.00	100.00		100.00
	200	100.00	100.00	100.00		100.00	100.00	100.00		100.00	100.00	100.00		100.00
	250	100.00	100.00	100.00		100.00	100.00	100.00		100.00	100.00	100.00		100.00
	300	100.00	100.00	100.00		100.00	100.00	100.00		100.00	100.00	100.00		100.00
Narva	25	100.00	99.98	99.96	99.68	99.17	99.99	99.83	98.32	98.09	99.80	99.62	98.36	98.92
	50	100.00	100.00	100.00	100.00	100.00	100.00	100.00	99.99	100.00	100.00	100.00		99.96
	75	100.00	100.00	100.00	100.00	100.00	100.00	100.00	100.00	100.00	100.00	100.00		100.00
	100	100.00	100.00	100.00	100.00	100.00	100.00	100.00		100.00	100.00	100.00		100.00
	125	100.00	100.00	100.00		100.00	100.00	100.00		100.00	100.00	100.00		100.00
	150	100.00	100.00	100.00		100.00	100.00	100.00		100.00	100.00	100.00		100.00
	200	100.00	100.00	100.00		100.00	100.00	100.00		100.00	100.00	100.00		100.00
	250	100.00	100.00	100.00		100.00	100.00	100.00		100.00	100.00	100.00		100.00
	300	100.00	100.00	100.00		100.00	100.00	100.00		100.00	100.00	100.00		100.00

Table S6. GSI accuracy (%) of SNPs ranked using pairwise F_{ST} (dataset II) tested on 26 independent individual populations (DRYAD entry doi:10.5061/dryad.gm367, Bourret et al. 2012).

**Population**	**Country**	**Number of SNPs ranked by pairwise F_{ST}**						**Number of randomly chosen SNPs**					
		25	50	75	100	125	150	25	50	75	100	125	150
Tuloma	Russia	73.93	86.00	94.72	97.49	98.45	98.66	40.92	47.71	85.12	93.22	97.46	97.57
Tana	Russia	73.82	86.29	96.39	98.68	98.57	98.73	11.11	57.74	82.49	93.97	95.79	99.45
Severnaya Dvina Emtsa	Russia	89.84	98.08	99.96	99.98	100.00	100.00	50.88	89.33	95.46	99.12	99.72	100.00
Ponoi Lebyazhya	Russia	76.98	87.16	89.08	96.21	97.87	98.10	31.79	63.00	84.35	92.09	96.10	96.57
Varzuga Yapoma	Russia	83.62	91.87	93.84	96.19	96.61	98.09	51.64	68.81	89.63	94.18	97.77	97.18
Pongoma	Russia	92.49	97.65	99.79	99.96	100.00	100.00	48.01	87.81	98.21	99.61	99.53	99.98
Suma	Russia	96.34	98.45	99.90	99.99	100.00	100.00	48.28	98.81	99.78	100.00	99.99	100.00
Numedalslågen	Norway	67.68	80.60	91.80	97.45	98.12	98.72	19.83	64.28	79.43	91.43	94.28	97.14
Gaula	Norway	60.00	71.88	86.64	91.54	93.92	94.91	24.74	61.29	75.33	81.76	90.56	95.99
Lærdalselva	Norway	53.02	76.93	87.54	93.41	93.57	96.51	6.55	61.99	79.77	85.09	90.83	96.22
Dart	Great Britain	32.39	56.92	74.96	85.10	88.11	91.49	23.55	39.05	64.81	65.83	79.33	90.42
Blackwater	Great Britain	22.07	38.40	59.01	71.26	77.44	83.07	16.46	47.21	56.54	66.75	71.71	82.80
Dionard	Great Britain	19.90	37.69	58.94	69.10	81.05	84.02	13.17	47.48	64.41	64.63	84.06	84.37
Foyle	Great Britain	25.43	47.48	68.88	75.15	83.87	86.92	27.31	52.99	72.02	82.93	87.27	86.14
Moy	Great Britain	29.70	63.64	74.84	86.68	93.21	94.63	19.02	39.20	65.27	76.19	83.03	89.50
North Esk	Great Britain	25.15	37.56	61.18	66.00	77.87	82.11	14.62	36.31	46.15	57.03	73.27	79.72
Tweed	Great Britain	19.13	43.96	67.48	76.75	83.73	88.13	15.32	43.93	67.66	65.69	78.62	81.99
Loire	France	58.22	94.18	98.91	99.66	100.00	100.00	58.11	86.68	96.40	99.56	99.95	100.00
Cares	Spain	91.64	99.83	100.00	100.00	100.00	100.00	89.58	99.77	99.99	100.00	100.00	100.00
Piguena	Spain	47.11	79.26	97.92	99.56	99.99	100.00	40.89	84.10	97.46	99.00	99.80	99.74
Narcea	Spain	69.78	95.82	99.89	99.98	99.99	100.00	74.55	93.51	99.14	99.89	99.59	99.74
Selá	Iceland	69.64	95.21	99.39	98.95	99.81	99.99	47.54	92.88	98.41	99.61	99.86	99.97
Ölfusá	Iceland	76.85	94.45	99.18	99.70	99.86	99.84	68.14	93.16	97.99	99.97	99.78	99.90
Tornionjoki	Finland	87.03	98.23	99.76	99.80	99.99	99.93	42.78	96.27	94.23	98.97	99.22	99.65
Vindelälven	Finland	85.28	97.37	99.88	99.97	99.97	100.00	52.45	97.66	94.38	99.44	99.86	99.55
Kunda	Estonia	83.32	99.36	99.99	100.00	100.00	100.00	59.63	99.35	99.95	100.00	100.00	100.00
**Overall**		**61.94**	**79.01**	**88.46**	**92.25**	**94.69**	**95.92**	**38.34**	**71.16**	**84.01**	**88.69**	**92.98**	**95.14**

Table S7. Estimated number of SNPs required to achieve 80%, 90%, 95% and 98% overall correct assignments for independent dataset of 26 European Atlantic salmon populations.

	**pairwise F_{ST} (dataset II)**	**random**
80%	50	71
90%	82	101
95%	120	133
98%	163	163
